# Outcome of Bloodstream Infections Caused by Antibiotic-Resistant Bacteria: A 7-Year Retrospective Study at the University Hospital of Palermo, Italy

**DOI:** 10.3390/antibiotics14050464

**Published:** 2025-05-01

**Authors:** Luca Pipitò, Eleonora Bono, Chiara Vincenza Mazzola, Raffaella Rubino, Antonio Anastasia, Salvatore Antonino Distefano, Alberto Firenze, Giovanni M. Giammanco, Celestino Bonura, Antonio Cascio

**Affiliations:** 1 Department of Health Promotion, Mother and Child Care, Internal Medicine and Medical Specialties “G. D’Alessandro”, University of Palermo, 90127 Palermo, Italy; eleonora.bono@community.unipa.it (E.B.); chiaravincenza.mazzola@community.unipa.it (C.V.M.); alberto.firenze@unipa.it (A.F.); giovanni.giammanco@unipa.it (G.M.G.); celestino.bonura@unipa.it (C.B.); 2 Infectious and Tropical Disease Unit and Sicilian Regional Reference Center for the Fight Against AIDS, AOU Policlinico “P. Giaccone”, 90127 Palermo, Italy; raffaella.rubino@policlinico.pa.it (R.R.); antonio.anastasia@policlinico.pa.it (A.A.); 3 Antimicrobial Stewardship Team, AOU Policlinico “P. Giaccone”, 90127 Palermo, Italy; salvatoreantonino.distefano@policlinico.pa.it; 4 Microbiology and Virology Unit, AOU Policlinico “P. Giaccone”, 90127 Palermo, Italy

**Keywords:** antimicrobial resistance, antimicrobial stewardship, hospital epidemiology bacteria, bacteremia, bloodstream infection, *Acinetobacter baumannii*, *Klebsiella pneumoniae*

## Abstract

Background: Bloodstream infections (BSIs) are both a primary cause and a severe complication of hospitalization. This retrospective study aims to analyze the epidemiology of BSIs at the University Hospital of Palermo from 2018 to 2024. Methods: We conducted a single-center, retrospective, observational study at the University Hospital Paolo Giaccone in Palermo, analyzing microbiological data from blood cultures collected between 1 January 2018 and 31 December 2024. Results: A total of 6345 blood culture isolates from 2967 patients were analyzed. Bacteremia-related mortality per 1000 patients rose from 5.1% in 2018 to 10.5% in 2024. The most isolated pathogens were non-*aureus* staphylococci (39.7%), followed by *Klebsiella pneumoniae* (12.1%) and *Staphylococcus aureus* (7.47%). *Acinetobacter baumannii* and *Pseudomonas aeruginosa* were more prevalent in ICUs. The number of *K. pneumoniae*, *A. baumannii*, *S. aureus*, and *P. aeruginosa* isolates per 1000 admitted patients increased significantly over time. Oxacillin resistance in *S. aureus* peaked at 49.0% in 2020 before declining, while among non-*aureus* staphylococci, it remained consistently high (>80%). Carbapenem-resistant *K. pneumoniae* peaked at 80% in 2022 before decreasing in 2024. Resistance to ceftazidime-avibactam and meropenem-vaborbactam was observed in 11.3% and 11.8% of *K. pneumoniae*, respectively. Multivariable analysis identified *A. baumannii* and *K. pneumoniae* BSIs as independent predictors of in-hospital mortality. Additionally, female sex, pneumonia, and central nervous system infections were significant risk factors for mortality. Conclusions: We observed an increasing trend in overall bacteremia-related mortality from 2018 to 2024. Microbiological data highlight the predominance of non-*aureus* staphylococci, *K. pneumoniae*, and *S. aureus* as leading pathogens of BSI, with *A. baumannii* emerging as a significant threat, particularly in ICUs. Rising antimicrobial resistance, especially among *K. pneumoniae*, underscores the urgent need for robust antimicrobial stewardship programs. *K. pneumoniae* and *A. baumannii* were associated with higher mortality.

## 1. Introduction

To optimize antimicrobial therapy, choosing the most suitable treatment and ensuring its prompt administration is essential. This task is especially difficult in hospital environments, where antimicrobial resistance (AMR) significantly hinders the selection of effective treatments. The silent pandemic of AMR represents a critical global health challenge. The World Health Organization (WHO) estimates that over one million deaths annually are directly attributable to AMR, with mortality rates expected to rise in the coming years [1]. Furthermore, the development of new antibiotics has been limited, and therapeutic options remain scarce for many of the WHO’s critical priority pathogens [2]. Epidemiological studies are essential for monitoring the evolution of AMR. At the same time, local surveillance is crucial for guiding appropriate empirical antibiotic therapy in hospitalized patients with infections before susceptibility testing results are available. Bloodstream infections (BSIs) are both a primary cause and a severe complication of hospitalization. They are also a significant cause of mortality, particularly among patients with terminal medical conditions, such as advanced malignancies or end-stage renal or hepatic disease [3]. A previous review estimated over 600,000 BSI episodes per year in North America, resulting in more than 90,000 deaths, while Europe records approximately 1,200,000 cases annually, with 157,000 associated deaths [3].

In 2023, the European data reported a decline in methicillin-resistant *S. aureus* (MRSA) bloodstream infections to 4.64 per 100,000 population, a 17.6% decrease from 2019. However, carbapenem-resistant *Klebsiella pneumoniae* infections increased by 57.5% since 2019, reaching 3.97 per 100,000 population. Increases in the estimated European incidences of bloodstream infections with resistant bacteria were observed also for vancomycin-resistant *Enterococcus faecium* (VRE) and piperacillin-tazobactam-, ceftazidime-, and carbapenem-resistant *Pseudomonas aeruginosa* [4]. A study in China reported that 15.1% of deceased patients had experienced a BSI within two weeks before death. The most frequently isolated pathogens were *K. pneumoniae*, *Escherichia coli*, and *S. aureus*, with a high prevalence of inappropriate empirical antimicrobial therapy in hospital-acquired BSIs [5]. Despite the significant mortality burden of BSIs [3], comprehensive data on the specific pathogens responsible for fatal outcomes and their clinical impact remain scarce. Understanding the epidemiology and antimicrobial susceptibility patterns of BSIs is essential for improving infection control strategies and optimizing antibiotic stewardship. According to the latest data from the Italian National Institute of Health (Istituto Superiore di Sanità, ISS), antibiotic resistance rates among major bacterial pathogens in Italy remain high, though some improvements have been observed. The proportion of MRSA isolates decreased to 26.6% in 2023, continuing a downward trend from 30% in 2021–2022, while there is a concerning increase in vancomycin-resistant VRE, with resistance rates rising from 11.1% in 2015 to 32.5% in 2023 [6]. For *E. coli,* resistance to third-generation cephalosporins slightly increased to 26.7% in 2023 from 24.2% in 2022. Additionally, after previous declines, resistance to aminoglycosides and fluoroquinolones rose to 14.5% and 34.1%, respectively, in 2023. For *K. pneumoniae,* carbapenem resistance rates increased to 26.5% in 2023 from 24.9% in 2022, and for *Acinetobacter* spp., resistance to major antibiotic classes remains high [6]. *P. aeruginosa* exhibited a slight improvement in its antibiotic resistance trend; specifically, carbapenem resistance was 16.0% in 2023. However, carbapenem resistance follows a clear north–south gradient, with higher resistance rates in southern regions compared to the north [6].

Furthermore, in Italy, including Sicily, there has been an increase in metallo-β-lactamase (MBL)-producing strains, with NDM being the most commonly detected [7]. To monitor bacterial resistance, the Sicilian Region established a microbiology laboratory network [8]. This initiative aims to increase the participation of regional microbiology laboratories in the national antibiotic resistance surveillance system, following European Centre for Disease Prevention and Control protocols [8,9]. Antibiotic resistance contributes significantly to treatment failure, relapsing infections, and increased healthcare burden [10]. A recent review showed that the emergence of carbapenem resistance in Gram-negative bacteria is a major global concern and involves new drugs. In particular, ceftazidime avibactam (CZA) resistance was highest in Asia, 19.3%, followed by Africa, 13.6%, Europe, 11%, South America, 6.1%, and North America, 5.3% [11]. Furthermore, AMR was associated with increased mortality, particularly in cases involving specific high-risk strains, such as CZA-resistant KPC-producing *Klebsiella pneumoniae* and *Acinetobacter baumannii* [12,13]. The latter has been linked to a mortality rate ranging from 30% to 75% [13]. Another work showed that MBL infections were associated with prolonged hospital/ICU stays and high mortality. In the latter study, of 6620 MBL-producers identified, 58.0% were from Europe and 31.4% from Asia. NDM (primarily NDM-1) was the predominant MBL (85.6%), while IMP and VIM producers were regionally concentrated in Asia and Europe, respectively [14].

This retrospective study aims to analyze the epidemiology of BSIs at the University Hospital of Palermo from 2018 to 2024, identify the pathogens associated with mortality, compare species and their antimicrobial resistance trends, and categorize bloodstream isolates according to the hospital wards where the infections were diagnosed.

## 2. Results

### 2.1. Study Population and Mortality

**Figure 1:**
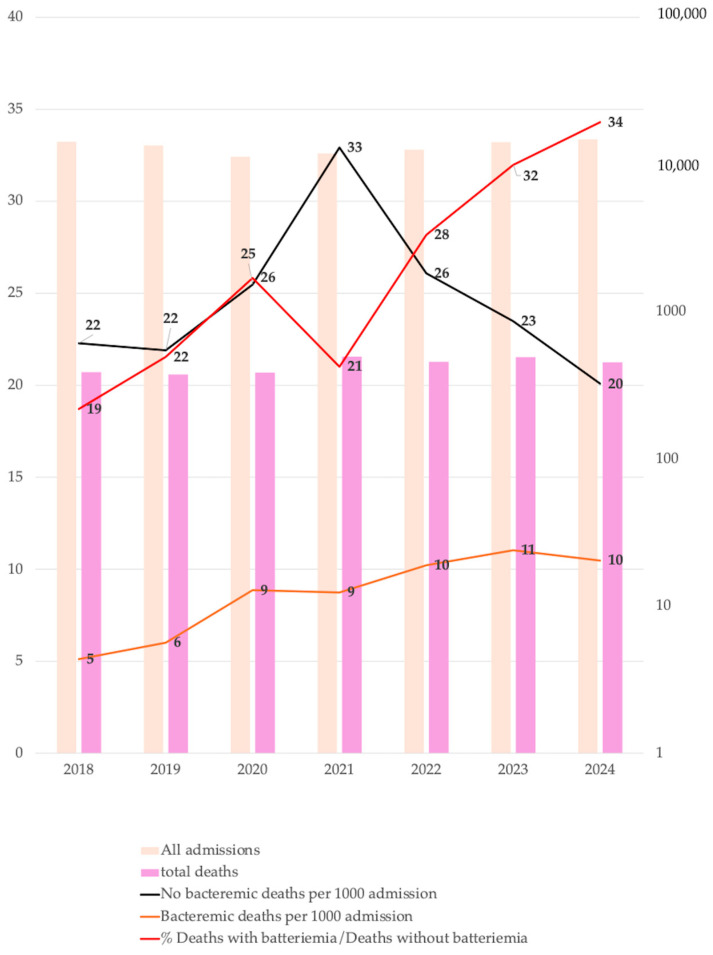
Number of hospital admissions and deaths per year, deaths for 1000 admissions with and without bacteremia, and percentage of deaths with bacteremia/all deaths.

During the study period, 6345 blood culture isolates were analyzed; 68.4% were obtained from peripheral veins, while the remaining samples were from central venous catheters. These isolates were derived from 2967 patients, of whom 59.0% were male, with a median age of 69 years (IQR 59–78). Women were significantly older than men (64.0 years vs. 66.1 years, *p* = 0.003). At the time of sample collection, 63.3% of patients were hospitalized in medical wards, 17.5% in intensive care units (ICUs), 17.0% in surgical wards, and 2.7% in neonatal ICUs (NICUs). Medical wards remained the most involved area, while ICUs and Surgery showed variations over time. The overall median length of stay (LOS) was 24 days (IQR 10–71). By ward, the median LOS was 22 days (IQR 12–36) for medical wards, 28 days (IQR 15–47) for surgical wards, 28 days (IQR 15–49) for ICUs, and 38 days (IQR 11–68.5) for NICUs. In-hospital mortality was 26.9%. The percentage of deaths with bacteremia among all deaths increased significantly over the years (β = 2.497, CI: 1.261–3.733, *p* = 0.003), and the deaths with bacteremia per 1000 patients increased from 5.1% in 2018 to 10.5% in 2024 (β = 0.982, CI: 0.548–1.416, *p* = 0.002). In 2021, during the COVID-19 pandemic, a peak of overall in-hospital mortality with 41.7 deaths per 1000 patients was observed. The data on yearly mortality are illustrated in Figure 1.

**Figure 2:**
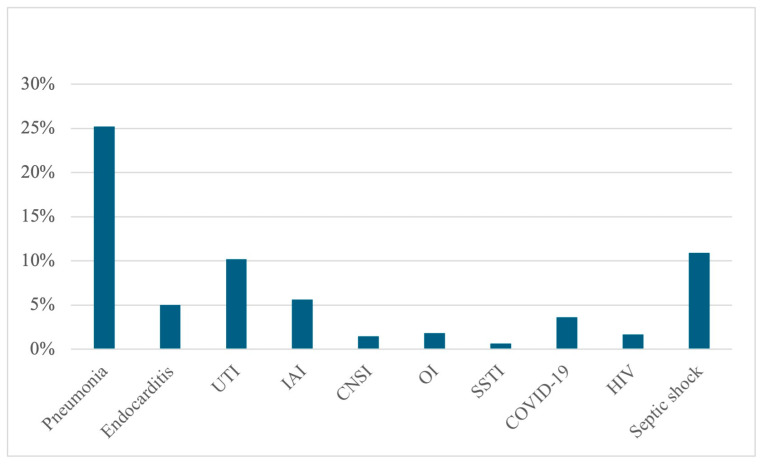
Prevalence of associated infections among 2967 patients. Pneumonia (25.2%), endocarditis (5.0%), UTI (10.2%), IAI (5.6%), CNSI (1.5%), OI (1.8%), SSTI (0.6%), COVID-19 (3.6%), HIV (1.7%), septic shock (10.9%). UTI: urinary tract infection; IAI: intra-abdominal infection; CNSI: central nervous system infection; OI: osteoarticular infection; SSTI: skin and soft tissue infection.

Mortality by ward was 17.04% in medical wards, 6.4% in surgical wards, 84.6% in ICUs, and 8.6% in NICUs. Among the 799 deceased patients, 320 were from medical wards (40.0%), 31 from surgical wards (3.9%), 441 from ICUs (55.2%), and 7 from NICUs (0.9%). BSIs were associated with a diagnosis of pneumonia in more than 25% of cases during hospitalization. The prevalence of all associated infections is depicted in Figure 2.

**Figure 3:**
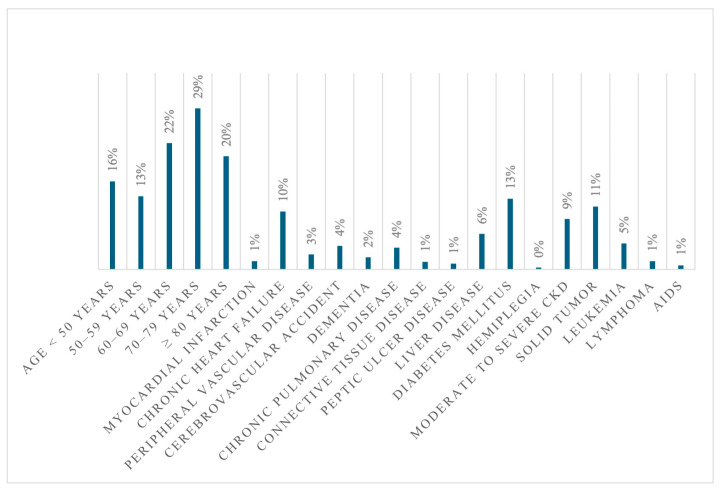
Prevalence of comorbidities and age distribution according to the Charlson Comorbidities Index.

The median Charlson Comorbidity Index (CCI) was 3 (IQR 2–5), with diabetes being the most common comorbidity (Figure 3).

**Figure 4:**
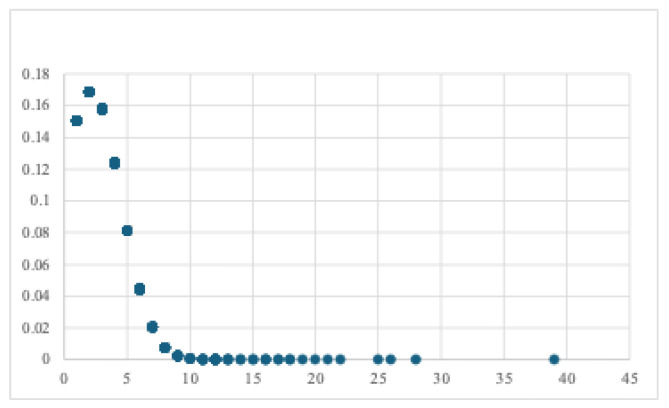
The scatter plot illustrates the relationship between the number of isolates (x-axis) and their prevalence in decimal per patient values (y-axis).

Multiple isolates could be found from a single patient, and the distribution of isolates per patient is shown in Figure 4. The mean number of isolates per patient varied significantly by ward (*p* < 0.001), with ICU patients exhibiting the highest number of isolates (mean: 3.53, CI: 3.18–3.88), followed by Medicine (1.86, CI: 1.78–1.94), Surgery 1.78, CI: 1.65–1.92), and NICU (1.69, CI: 1.42–1.96). The number of isolates increased significantly in association with LOS (ß = 0.041, CI: 0.038, 0.043, *p* < 0.001).

Univariate analysis for in-hospital mortality identified a significant association with ICU admission, septic shock, pneumonia, central nervous system infections, and COVID-19 (Table 1).

### 2.2. Microbiological Isolates

**Figure 5:**
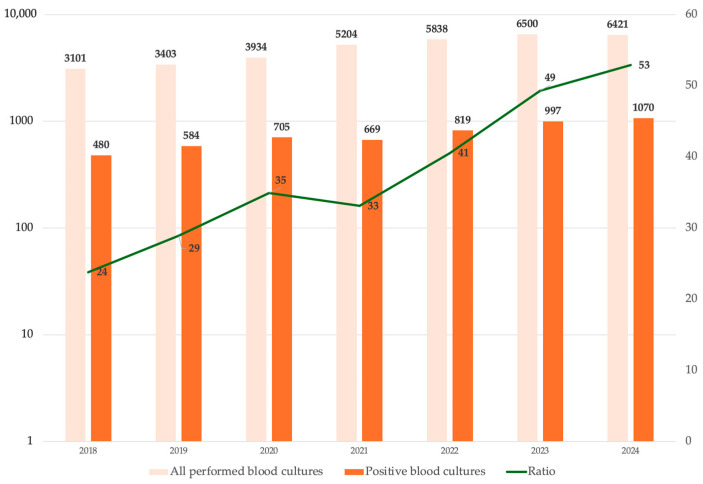
Total number of blood cultures performed (light orange bars) and positive blood cultures (dark orange bars) from 2018 to 2024, plotted on a semi-logarithmic scale. The green line represents their ratio, showing a significant increasing trend of positive blood cultures over time.

After removing duplicate isolates, a total of 5324 unique isolates were analyzed, distributed as follows: 501 in 2018, 580 in 2019, 733 in 2020, 624 in 2021, 819 in 2022, 1078 in 2023, and 989 in 2024. The annual prevalence of blood isolates per total admissions ranged from 16.2% in 2018 to 15.4% in 2024, showing no significant trend. In contrast, the prevalence of positive blood cultures among all blood cultures performed increased significantly (ß = 4.77, CI: 3.49, 6.04, *p* < 0.001), as shown in Figure 5.

**Figure 6:**
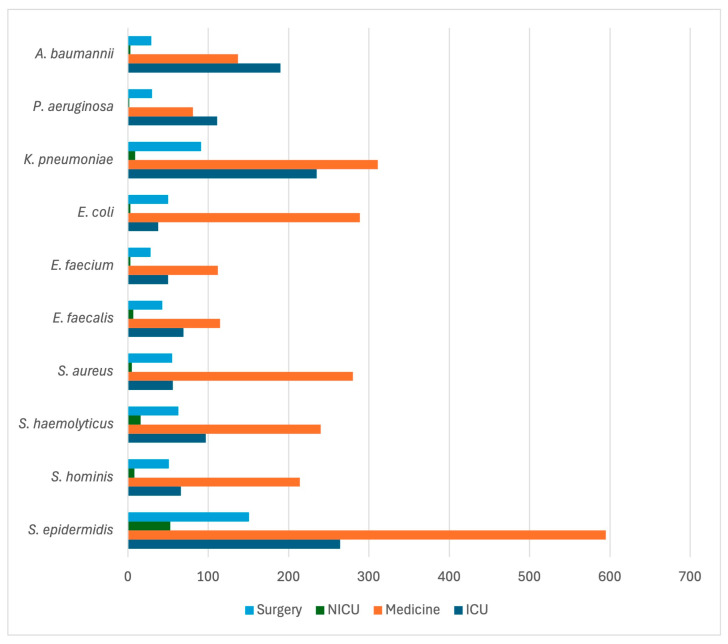
Absolute frequency of the first ten isolates across different wards (medical, surgical, ICU, and NICU).

The most frequently isolated pathogens were non-*aureus* staphylococci (39.7%), followed by *K. pneumoniae* (12.1%) and *S. aureus* (7.47%). The prevalence of each pathogen by ward is detailed in Table 2. The predominant isolates per ward are depicted in Figure 6. While non-*aureus* staphylococci, *K. pneumoniae*, *Enterococcus* spp., and *S. aureus* were most commonly isolated from medical wards, *A. baumannii* and *P. aeruginosa* were more prevalent in ICUs.

**Figure 7:**
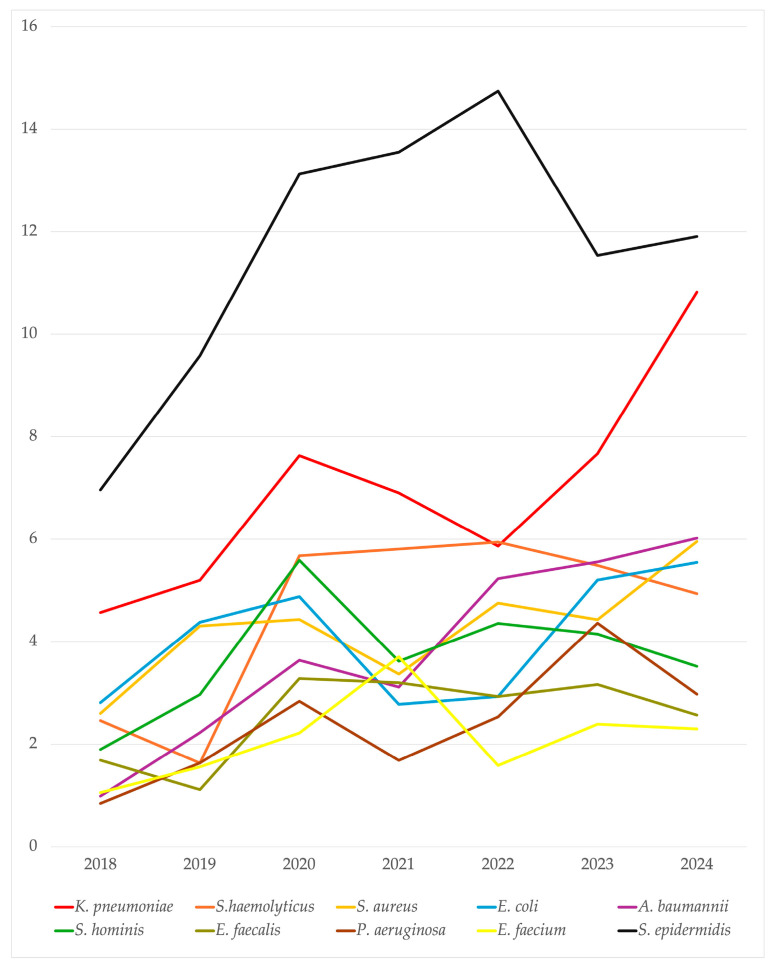
Distribution of the 10 most common isolates per 1000 admitted patients.

*A. baumannii* (R = 0.253) and *P. aeruginosa* (R = 0.112) exhibit the highest positive correlation with ICU settings (*p* < 0.001). *K. pneumoniae* (R = 0.07270) also exhibits a mild correlation with ICUs (*p* < 0.001). *E. coli* (R = 0.100) is the most strongly correlated pathogen with medical wards (*p* < 0.001). The number of isolated microorganisms per 1000 admitted patients is illustrated in Figure 7. Distributions of isolates per 10,000 blood cultures and 1000 patient days are depicted in Supplementary Figures S1 and S2. Over the study period, the prevalence of *A. baumannii* isolates increased significantly (ß = 0.551, CI: 0.156–0.946, *p* = 0.016); whereas, no significant trends in prevalence were observed for *K. pneumoniae*, *Staphylococcus* spp., *Enterococcus* spp., *P. aeruginosa*, or *Enterobacter* spp. However, the number of *K. pneumoniae* (ß = 0.783, CI: 0.142, 1.425, *p* = 0.025), *A. baumannii* (ß = 0.834, CI: 0.571, 1.097, *p* < 0.001), *S. aureus* (ß = 0.379, CI: 0.023, 0.736, *p* = 0.041), and *P. aeruginosa* (ß = 0.412, CI: 0.027, 0.800, *p* = 0.040) isolates per 1000 admitted patients increased significantly.

*E. coli* showed a positive correlation with urinary tract infection (R = 0.17570) and *S. aureus* with endocarditis (R = 0.11206) and osteoarticular infections (R = 0.11981), *p* < 0.001.

A total of 638 bloodstream infections were exclusively associated with central venous catheters (CVCs), involving 442 patients, with *S. epidermidis* being the most frequently isolated pathogen (Supplementary Figures S3 and S4). Patients with CVC-related infections had a significantly longer LOS compared to those without CVC infections (55.5 ± 46.4 days vs. 40.7 ± 38.4 days, *p* < 0.001) as well as a higher mortality rate (43.8% vs. 26.4%, *p* < 0.001).

### 2.3. Antimicrobial Resistance

Data on the prevalence of antimicrobial resistance are summarized in Table 3.

**Figure 8:**
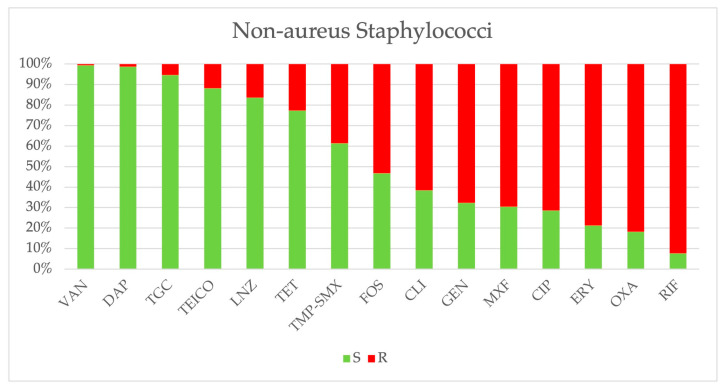
Cumulative antibiograms of 2113 non-*aureus* staphylococci isolated from 2018 to 2024. VAN: vancomycin, DAP: daptomycin, TGC: tygeciclin, OXA: oxacillin, TEICO: teicoplanin, LNZ: linezolid, TET: tetracycline, TMP-SMX: trimethoprim-sulfamethoxazole, FOS: fosfomycin, CLI: clindamycin, GEN: gentamicin, MXF: moxifloxacin, CIP: ciprofloxacin, ERY: erythromycin, RIF: rifampicin.

**Figure 9:**
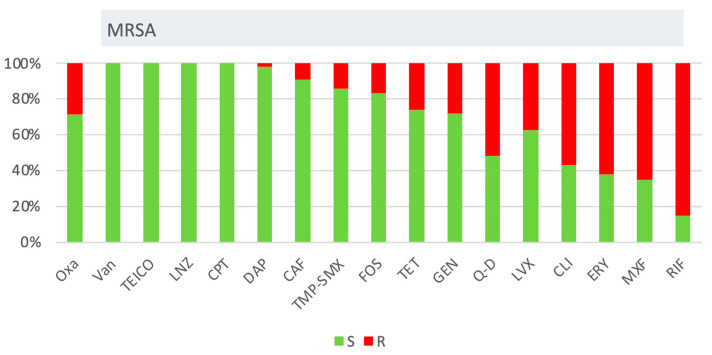
Cumulative antibiograms of 117 MRSA strains isolated from 2018 to 2024. The first column refers comprehensively to all the strains of *S. aureus*, while the others refer only to MRSA. Oxa: oxacillin, Van: vancomycin, TEICO: teicoplanin, LNZ: linezolid, CPT: ceftaroline, DAP: daptomycin, CAF: chloramphenicol, TMP-SMX: trimethoprim-sulfamethoxazole, FOS: fosfomycin, TET: tetracycline, GEN: gentamicin, Q-D: quinupristin-dalfopristin, LVX: levofloxacin, CLI: clindamycin, ERY: erythromycin, MXF: moxifloxacin, RIF: rifampicin.

*Staphylococcus* spp. Oxacillin resistance among non-*aureus* staphylococci remained high (>80%) (Figure 8). Fosfomycin resistance significantly increased (from 27.9% in 2018 to 63.1% in 2023, β = 5.336, CI: 2.065–8.607, *p* = 0.009), while oxacillin resistance in *S. aureus* declined from 13.9% to 3.7% (*p* = 0.077). MRSA prevalence fluctuated, peaking at 49.0% in 2020, subsequently decreasing (*p* = 0.119). Ciprofloxacin resistance declined from 47.4% in 2018 to 22.5% in 2024 (β = −4.154, CI: −6.869–−1.438, *p* = 0.011). Vancomycin resistance remained low (<1.5%) among all staphylococcal species. Comparative analysis between MRSA (*n* = 117) and methicillin-susceptible *S. aureus* (MSSA, *n* = 281) revealed significantly higher resistance rates in MRSA strains. MRSA exhibited greater resistance to gentamicin (27.8% vs. 10.3%, *p* < 0.001, OR 3.35, CI: 1.91–5.7), ciprofloxacin (66.1% vs. 11.4%, *p* < 0.001, OR 15.16, CI: 8.89–25.85), TMP-SMX (12.2% vs. 9.8%, *p* < 0.001, OR 7.62, CI: 2.68–21.70), tetracycline (26.1% vs. 5.7%, *p* < 0.001, OR 5.85, CI: 3.04–11.24), fosfomycin (17.4% vs. 2.5%, *p* < 0.001, OR 8.18, CI: 3.35–19.96), clindamycin (*p* < 0.001, OR 3.96, CI: 2.51–6.26), moxifloxacin (65.8% vs. 10.3%, *p* < 0.001, OR 16.76, CI: 9.67–29.05), and erythromycin (62.6% vs. 26.3%, *p* < 0.01, OR 4.68, CI: 2.95–7.43). Similarly, non-*aureus* staphylococci (*n* = 2054) displayed higher resistance to multiple antibiotics, including oxacillin, gentamicin, ciprofloxacin, TMP/SMX, tetracycline, fosfomycin, erythromycin, teicoplanin, linezolid, clindamycin, and moxifloxacin, compared to *S. aureus* (*p* < 0.001). No ceftaroline-resistant (0/117) *S. aureus* isolates were detected. The cumulative antibiogram of MRSA is depicted in Figure 9.

*K. pneumoniae.* The prevalence of carbapenem-resistant *K. pneumoniae* fluctuated, peaking at 80% in 2022 and decreasing to 58–70% in 2024 (*p* = 0.650). Cefepime and ceftazidime resistance remained high (84–90%); whereas, colistin resistance declined from 16.5% in 2021 to 0.7% in 2024 (*p* = 0.542). Among carbapenem-resistant *K. pneumoniae* isolates, higher resistance was observed for gentamicin (72.0% vs. 45.4%, *p* < 0.001), amikacin (26.7% vs. 3.4%, *p* < 0.001), ciprofloxacin (99.8% vs. 54.2%, *p* < 0.001), fosfomycin (39.9% vs. 11.4%, *p* < 0.001), and colistin (9.5% vs. 2.7%, *p* = 0.002) compared to carbapenem-susceptible strains. Resistance to CZA and meropenem-vaborbactam was 11.3% and 11.8%, respectively.

**Figure 10:**
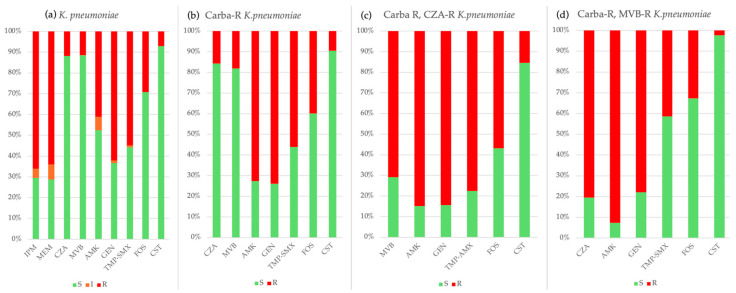
Cumulative antibiogram of 646 *K. pneumoniae* strains isolated from 2018 to 2024: (**a**) all strains, (**b**) carbapenem-resistant strains, (**c**) ceftazidime-avibactam-resistant strains, (**d**) meropenem-vaborbactam-resistant strains. IPM: imipenem, MEM: meropenem, CZA: ceftazidime-avibactam, MVB: meropenem-vaborbactam, AMK: amikacin, GEN: gentamicin, TMP-SMX: trimethoprim-sulfamethoxazole, FOS: fosfomycin, CST: colistin.

Strains of *K. pneumoniae* resistant to CZA exhibited a higher prevalence of resistance to colistin (15.5% vs. 2.4%), fosfomycin (56.9% vs. 48.8%), and TMP-SMX compared to those resistant to meropenem-vaborbactam (74.1% vs. 70.7%). Among CZA-resistant strains, the prevalence of meropenem-vaborbactam resistance was 68.75% (33/48). Conversely, meropenem-vaborbactam-resistant strains demonstrated a higher rate of resistance to amikacin compared to CZA-resistant strains (85.4% vs. 53.4%), with the prevalence of CZA resistance among meropenem-vaborbactam-resistant strains reaching 80.5% (33/41). The cumulative antibiogram of *K. pneumoniae* strains resistant to meropenem, ceftazidime-avibactam, and meropenem-vaborbactam, isolated from 2018 to 2024, is illustrated in Figure 10.

*A. baumannii* AMR remained critically high (80–100%) for all antibiotics except colistin. Cefiderocol susceptibility testing (*n* = 114) found no resistant isolates, including 70 *A. baumannii*, 41 *K. pneumoniae*, 2 *P. aeruginosa*, and 1 *E. coli*. For other *Acinetobacter* spp. (*n* = 19), the prevalence of resistance to various antibiotics was as follows: meropenem (5/19), gentamicin (4/19), ciprofloxacin (4/18), and trimethoprim-sulfamethoxazole (TMP-SMX) (6/19), with no resistance observed to colistin (0/16).

**Figure 11:**
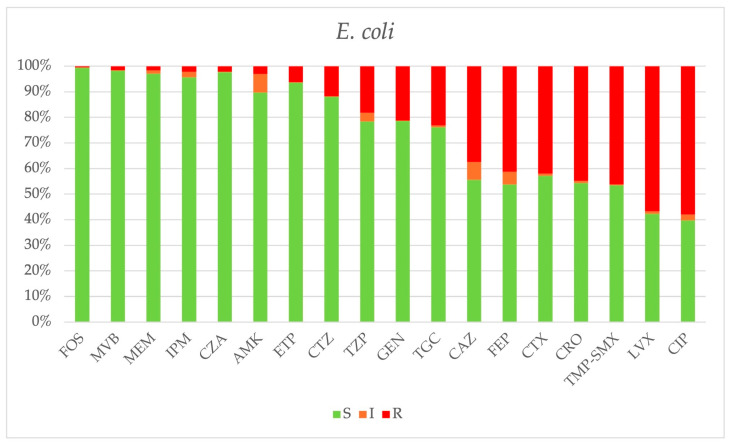
Cumulative antibiogram of 380 *E. coli* strains isolated from 2018 to 2024. FOS: fosfomycin, MVB: meropenem-vaborbactam, MEM: meropenem, IPM: imipenem, CZA: ceftazidime-avibactam, AMK: amikacin, ETP: ertapenem, CTZ: ceftolozane-tazobactam, TZP: piperacillin-tazobactam, GEN: gentamicin, TGC: tigecycline, CAZ: ceftazidime, FEP: cefepime, CTX: cefotaxime, CRO: ceftriaxone, TMP-SMX: trimethoprim-sulfamethoxazole, LVX: levofloxacin, CIP: ciprofloxacin.

*E. coli.* Carbapenem resistance remained low (<10%), while resistance to cefepime (*p* = 0.253) and ceftazidime (*p* = 0.340) showed a slight increase, ranging from 38% to 44%. Ciprofloxacin resistance remained high, ranging from 50% to 62%. The cumulative antibiogram of *E. coli* is depicted in Figure 11.

**Figure 12:**
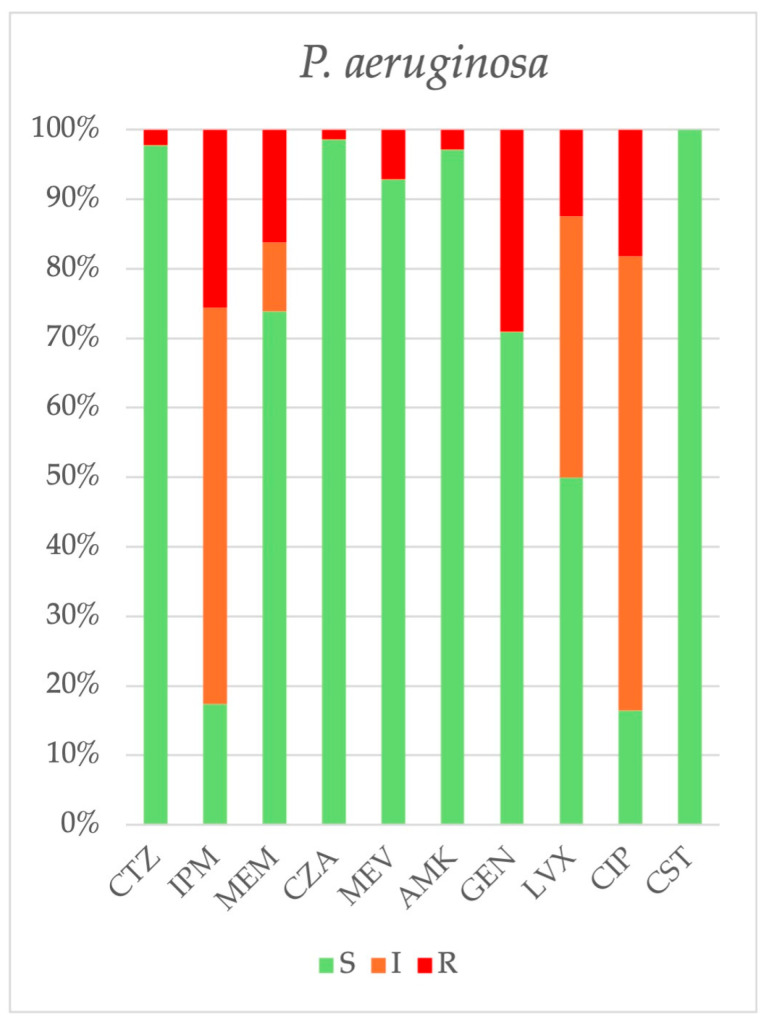
Cumulative antibiogram of 224 *P. aeruginosa* strains isolated from 2018 to 2024. CAZ: ceftazidime, IPM: imipenem, MEM: meropenem, CZA: ceftazidime-avibactam, MVB: meropenem-vaborbactam, AMK: amikacin, GEN: gentamicin, LVX: levofloxacin, CIP: ciprofloxacin, CST: colistin.

*P. aeruginosa.* Carbapenem resistance fluctuated but demonstrated a downward trend, decreasing from 58.3% in 2018 to 2.6% in 2024 (β = −7.239, CI: −11.904, −2.575, *p* = 0.010). Tobramycin resistance significantly declined from 66.7% in 2018 to 2.7% in 2024 (*p* = 0.060). Furthermore, carbapenem-resistant *P. aeruginosa* strains exhibited a higher prevalence of resistance to aminoglycosides than carbapenem-susceptible strains (44.4% vs. 5.4%, *p* < 0.001). The prevalence of resistance to ceftolozane-tazobactam remained low (2.2%). The cumulative antibiogram of *P. aeruginosa* strains isolated from 2018 to 2024 is shown in Figure 12.

Others. The prevalence of VRE steadily increased from 0% in 2018 to 53.1% in 2024 (β = 8.832, CI: 7.565–10.099, *p* < 0.001). For *Corynebacterium* spp. (*n* = 100), the resistance prevalence was as follows: penicillin (87/97), gentamicin (28/61), ciprofloxacin (90/98), and tetracycline (67/93). No resistance was detected to teicoplanin (0/50) or vancomycin (0/99), while resistance to linezolid was very low (1/92). For *Streptococcus* spp. (*n* = 96), resistance prevalence included: penicillin (4/92), vancomycin (1/96), erythromycin (13/49), cefotaxime (2/81), clindamycin (24/95), and clarithromycin (7/33). For *Serratia* spp. (*n* = 70), resistance prevalence was as follows: meropenem (0/69), gentamicin (2/69), ciprofloxacin (2/70), TMP-SMX (0/70), and fosfomycin (1/66). For *Proteus* spp. (*n* = 57), resistance was observed for piperacillin-tazobactam (2/57), cefepime (9/57), gentamicin (33/57), ciprofloxacin (37/57), TMP-SMX (37/57), and fosfomycin (27/57), while no resistance was detected to meropenem (0/56). For *Stenotrophomonas maltophilia* (*n* = 44), the prevalence of resistance was as follows: TMP-SMX (2/44) and levofloxacin (9/10), with no resistance observed to colistin (0/6).

### 2.4. Lethality

**Figure 13:**
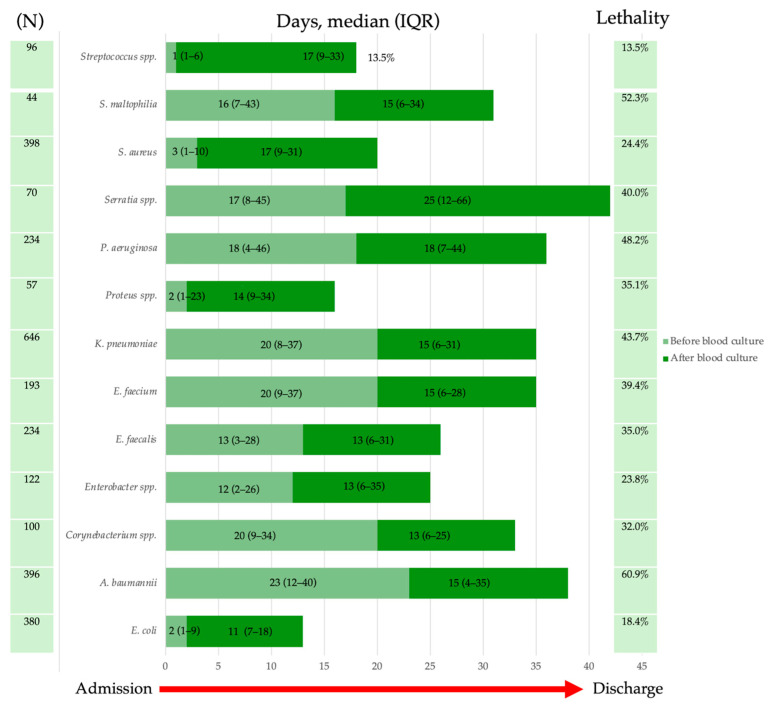
Duration of hospitalization for specific microorganisms (N = absolute frequency): median length of stay and interquartile range (IQR) before (light green) and after (dark green) blood culture, along with associated lethality.

**Figure 14:**
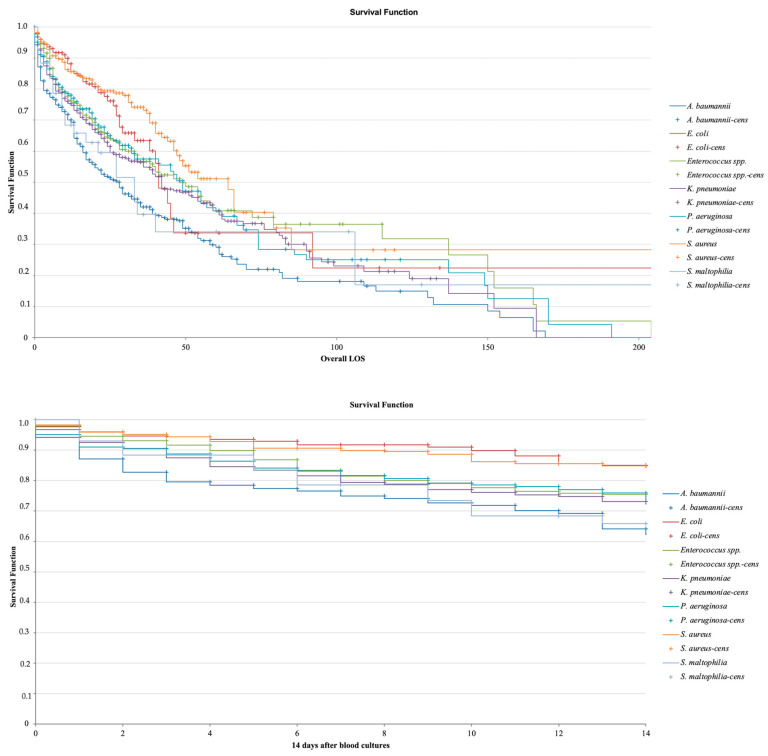
Kaplan–Meier survival analysis by pathogen: (above) overall LOS after blood culture collection, (below) survival at 14 days after blood culture collection.

Among deceased patients, the most frequently isolated pathogens were *K. pneumoniae* (15.5%) and *A. baumannii* (13.3%). However, *K. pneumoniae* exhibited a decreasing trend in prevalence from 26.0% in 2018 to 10.2% in 2023, with a modest resurgence to 16.3% in 2024. *A. baumannii* showed a notable increase from 8.7% in 2018 to 14.0% in 2019, maintaining a relatively stable prevalence (14%) in subsequent years. Other pathogens, including *P. aeruginosa*, *E. faecalis*, and *E. faecium*, displayed variable but moderate prevalence levels. *P. aeruginosa* peaked at 9.0% in 2023, while *E. faecalis* and *E. faecium* fluctuated between 3 and 7% throughout the study period. *E. coli* remained below 6% in all years. The lethality associated with blood culture positivity for specific pathogens during hospitalization across different hospital wards is represented in Table 4. A higher lethality was observed among patients hospitalized in ICUs. When comparing medical and surgical wards, lethality was higher in medical wards for all major isolates except *P. aeruginosa*, for which lethality was lower in medical wards compared to surgical wards (17.3% vs. 23.3%). Lethality was higher among ICU patients compared to other wards. Figure 13 illustrates the LOS before and after a positive blood culture for specific pathogens and associated mortality. The median LOS before blood culture ranged from 1 (IQR 1–6) for *Streptococcus* spp. to 23 (IQR 12–40) for *A. baumannii*, while after blood culture, the median LOS ranged from 11 (IQR 1–9) for *E. coli* to 25 (IQR 12–66) for *Serratia* species. Patients with *S. pneumoniae, S. aureus,* and *E. coli* had a shorter LOS before blood cultures were performed, likely because these pathogens are primarily community-acquired. Survival analysis (Figure 14) revealed significantly reduced survival in patients with BSIs caused by *A. baumannii*, *S. maltophilia*, and *K. pneumoniae* (*p* < 0.001). *A. baumannii* bacteremia was associated with significantly lower survival rates than *K. pneumoniae* (*p* < 0.001), at 14 days from blood culture collection and throughout the entire hospitalization. In contrast, no difference was observed between *A. baumannii* and *S. maltophilia* (*p* = 0.604). Figure S5 illustrates survival curves for all isolates with an absolute frequency > 50. Multivariable analysis identified *A. baumannii* and *K. pneumoniae* bloodstream infections as independent predictors of in-hospital mortality, as shown in Table 5. Additionally, pneumonia and central nervous system infections were significant risk factors for mortality.

## 3. Discussion

We found a rising mortality trend in patients diagnosed with bacteremia from 2018 to 2024. The mortality exceeded 80% in ICU patients. However, the attributable mortality of bacteremia, based on the study’s methodology, cannot be stated with certainty. In a 12-year population-based cohort study conducted by Nielsen et al. involving 7783 patients, bacteremia was associated with a poor prognosis and a considerable excess in long-term, all-cause mortality compared to the general population [15]. In 2021, the ratio of bacteremia-related mortality to overall mortality decreased, likely due to the increased mortality associated with COVID-19, which was also identified as a predictor of in-hospital mortality in our univariate analysis. Previous studies have reported an increase in the incidence of bacteremia during the COVID-19 pandemic. Cauhapé et al. observed that the incidence of bacteremia rose from 9.7 per 1000 h in 2019 to 19.7 per 1000 h in 2020 [16]. Similarly, Bauer et al., in a multicenter retrospective study, found higher rates of hospital-onset BSIs during the pandemic compared to the pre-pandemic period (2.0 vs. 1.5 per 1000; *p* < 0.001), with an increased detection of *Enterococcus* spp., *Staphylococcus aureus*, *Klebsiella pneumoniae*, and *Candida albicans* [17]. A Spanish study also reported a substantial increase in hospital-acquired catheter-related bacteremia cases in 2020 compared to the predicted rate based on data from 2007 to 2019 [18]. In our data, we did not observe significant peaks in the prevalence of specific blood isolates during the COVID-19 pandemic. However, our previous study reported a peak in the isolation of ESKAPE microorganisms across all kinds of samples in 2021 [19]. Notably, we observed a significant increase in the prevalence of positive blood cultures among all performed blood cultures and a rising number of *K. pneumoniae*, *A. baumannii*, *S. aureus*, and *P. aeruginosa* isolates per 1000 admitted patients over the years. The first finding may be attributed to antimicrobial stewardship programs promoting the targeted execution of blood cultures. The number of blood isolates per patient increased proportionally with LOS and was associated with higher mortality in the univariate analysis; however, this association was not confirmed in the multivariable analysis. Patients admitted to ICUs had a higher number of blood isolates than those in medical and surgical wards. *A. baumannii* and *P. aeruginosa* showed a significant positive correlation with ICUs, while *E. coli* shows this with medical wards. The diagnoses of pneumonia and central nervous system infections were linked to increased mortality in patients with BSIs. However, it remains unclear whether these infections preceded, followed, or were directly associated with bacteremia. A previous study assessing mortality risk factors in patients with *A. baumannii* bacteremia reported high early mortality (61.5%), with severity of illness being the only independent predictor of early mortality [20]. Mortensen et al. found that hospital-acquired bacteremia was associated with increased mortality, particularly when the source was unknown, pneumonia-related, or of abdominal origin [21]. Singh et al. found high rates of *K. pneumoniae*, *A. baumannii*, and *P. aeruginosa* isolates in severe pneumonia patients admitted to the ICU, showing that sepsis, ventilator associated pneumonia, and underlying cerebrovascular disease were the most common factors associated with carbapenem resistance, and that the 30-day survival rate was lower among patients infected with multiple pathogens [22]. The UNIFORM study identified factors influencing bacteremia mortality, including baseline patient characteristics, infection acquisition setting, infection-specific variables, inflammatory response at the onset of sepsis, and management parameters [23]. García Rodríguez et al., in a large retrospective cohort study, reported that male sex, advanced age, immunosuppressive therapy, and polymicrobial bacteremia were associated with higher mortality. Interestingly, consistent with our findings, endocarditis was linked to lower mortality, likely due to the benefits of early and appropriate therapy [24]. However, we cannot definitively determine whether patient mortality was directly attributable to bacteremia. In contrast to previous results [24], considering a mild major female age, we highlight a significantly higher mortality in the female sex, which was confirmed in multivariable analysis. Microbiological analysis revealed that non-*aureus* staphylococci were the most commonly isolated microorganisms in blood cultures (39.7%). While coagulase-negative staphylococci (CoNS) are increasingly recognized as significant nosocomial pathogens, particularly in bloodstream infections related to indwelling catheters and prosthetic heart valves [25,26], their pathogenic role remains uncertain in many cases, and a large proportion were likely contaminants. *S. epidermidis* was the most frequently isolated species from CVCs. According to our findings, a monocentric study from Colombia demonstrated a positive association between central line-associated bloodstream infections, increased mortality risk, and prolonged hospital stays. While we identified non-*aureus* staphylococci as the most frequently involved pathogens, their study reported *K. pneumoniae* and *S. aureus* as the predominant isolates [27]. Another study reported an overall 30-day mortality rate of 33.6% in patients with central line-associated BSIs, which is lower than our results (43.8%) [28]. However, in our study, higher mortality in patients with CVC infections is likely attributable to their frequent admission to the ICU or their need for dialysis, both of which are associated with greater disease severity and worse clinical outcomes. According to the 2022–2023 ECDC report, CoNS accounted for 16.4% of blood isolates in Europe [29]. Our data revealed a higher prevalence of CoNS (39.7%), which may reflect suboptimal blood culture collection practices. Additionally, medicine wards showed a higher prevalence of CoNS compared to ICUs, likely due to less stringent adherence to aseptic protocols during blood culture collection in these wards. The latest AMCLI (Associazione Microbiologi Clinici Italiani) recommendations, based on recent literature, provide a single-sample strategy for collecting blood cultures via a single venipuncture [30]. However, the Infectious Diseases Society of America and the American Society for Microbiology suggest collecting by separate venipunctures, one after the other [31]. Collecting blood cultures from the same vein at the same time increases the risk of misinterpreting CoNS or other microorganisms as true pathogens rather than contaminants. In fact, microorganisms introduced due to inadequate skin disinfection may grow in multiple culture bottles, leading clinicians to suspect a pathogenic role. Excluding staphylococci non-aureus isolates, *K. pneumoniae* and *S. aureus* emerged as the most frequently identified pathogens. *A. baumannii* was predominantly isolated in ICUs, with its prevalence increasing significantly over time. A previous study in Shanghai identified *A. baumannii* as the predominant ICU-acquired species, exhibiting higher carbapenem resistance than isolates present during ICU admission. The reported carbapenem resistance rate (23.8–44.8%) was significantly lower than our findings [32]. A Hungarian study on ESKAPE bacteria identified *E. coli* as the most frequent isolate, followed by the *Klebsiella* genus and *S. aureus* (MRSA 19.6%) [33]. A multicenter study in Taiwan (2002–2020) identified *E. coli* (31%), *S. aureus* (13.6%), and *K. pneumoniae* (12.7%) as the most common pathogens causing bacteremia [34]. In our study, antimicrobial resistance analysis revealed a decline in oxacillin resistance among *S. aureus*; whereas, non-*aureus* staphylococci exhibited high resistance rates (>80%). Vancomycin resistance remained low among *Staphylococcus* spp. However, it increased significantly in *E. faecium* (from 0% in 2018 to 53.1% in 2024). Notably, non-*aureus* staphylococci exhibited discordant resistance patterns to teicoplanin compared to their susceptibility to vancomycin (11.8% vs. 0.6%), previously described in CoNS [25]. MRSA strains exhibited high resistance rates to most tested antibiotics. While *S. aureus* and non-*aureus* staphylococci demonstrated low resistance to daptomycin (1.2–15%), the latter exhibited higher resistance to linezolid (16.3%). Huang et al. reported an alarming increase in vancomycin resistance among *E. faecium* (10.0% in 2004 to 47.7% in 2020) [34]. A German review documented a declining trend in MRSA infections (7.9% between 2014 and 2020), while *E. faecium* bloodstream infections showed rising vancomycin resistance (34.9%) [35]. For *P. aeruginosa*, we observed a decline in meropenem resistance from 58.3% in 2018 to 2.6% in 2024, and ceftolozane-tazobactam maintained a very low resistance rate (2.2%). *K. pneumoniae* displayed high carbapenem resistance, peaking at 80% in 2022. Carbapenem-resistant strains also exhibited greater resistance to aminoglycosides, ciprofloxacin, and colistin than carbapenem-susceptible strains. A large U.S. hospital cohort study analyzing 41.6 million hospitalizations reported an overall rate of 292 clinical cultures per 1000 patient-days, revealing that MRSA and ESBL infections accounted for most infections [36]. Between 2012 and 2017, the incidence of MRSA, VRE, carbapenem-resistant *Acinetobacter* spp., and multidrug-resistant *P. aeruginosa* infections declined. In contrast, the incidence of carbapenem-resistant Enterobacteriaceae infections remained stable, while ESBL infections increased by 53.3%, driven primarily by a rise in community-onset cases [36]. Resistance to CZA and meropenem-vaborbactam among carbapenem-resistant *K. pneumoniae* was similar (11.3% vs. 11.8%). CZA-resistant strains exhibited higher resistance to colistin and amikacin than meropenem-vaborbactam-resistant strains. Resistance to CZA is an emerging nosocomial concern. CZA-resistant, KPC-variant *K. pneumoniae* strains have been reported in the literature [11,37,38,39]. A previous Italian study found that 10.5% of bloodstream infections caused by carbapenem-resistant *K. pneumoniae* between 2018 and 2022 were attributed to ceftazidime-avibactam-resistant strains [40]. Another Italian study analyzing 59 patients with *K. pneumoniae* infections reported that 57.6% had ceftazidime-avibactam-resistant strains [41]. Five cases of meropenem-vaborbactam-resistant *K. pneumoniae* bloodstream infections were reported in Northern Italy in 2018. Genomic analysis revealed that resistance was associated with a truncated OmpK35 and glycine and aspartic acid insertion within OmpK36 at positions 134–135 (GD134–135) [42]. Another Italian study (2018–2019) identified CZA resistance in three KPC-producing *K. pneumoniae* strains isolated from patients with *K. pneumoniae* BSIs who had no prior exposure to ceftazidime-avibactam-based therapy. Two of these strains were also resistant to meropenem-vaborbactam. The CZA-resistant strain harbored a mutated KPC-2 enzyme; whereas, the other two resistant strains exhibited non-functional *ompK35-ompK37* and mutated *ompK36* porins, along with an increased copy number of the *blaKPC* gene [43]. Additionally, the spread of metallo-β-lactamase-producing strains may contribute to the rising resistance to ceftazidime-avibactam and meropenem-vaborbactam [7]. However, our study lacks genetic analysis, which prevents us from drawing conclusions regarding the resistance mechanisms of *K. pneumoniae*. *A. baumannii* exhibited high resistance rates to all tested antibiotics, except for colistin. Cefiderocol emerged as a potential treatment option for *A. baumannii*, with no observed resistance among the tested isolates. A multicenter Greek study found cefiderocol to be effective against at least 86% of *A. baumannii* bloodstream infections [44]. In Taiwan, between 2018 and 2020, among 255 *A. baumannii* isolates, 94.9% were inhibited at <4 mg/L [45]. However, cefiderocol-resistant strains were identified, with resistance rates ranging from 24% to 33.3%, depending on the susceptibility testing method used [46]. For *E. coli*, ceftazidime resistance increased significantly over the study period, rising from 23.8% to 43.9%. Similarly, a multicenter study from Taiwan observed a notable increase in third-generation cephalosporin non-susceptibility among *E. coli* [34]. In a Greek study describing the epidemiology of ESKAPEE-associated bacteremia from 2016 to 2021, multidrug resistance was lowest for *P. aeruginosa* (30%) and *E. coli* (33%) but highest among *A. baumannii* (97%). Notably, *A. baumannii* bacteremia was associated with the highest mortality rate [47]. A 5-year Canadian surveillance study on Gram-negative antimicrobial resistance, encompassing all infection types (of which 15% were BSIs), reported *E. coli*, *K. pneumoniae*, and *P. aeruginosa* as the most frequently isolated pathogens. In contrast, our findings identified *K. pneumoniae*, *A. baumannii*, and *E. coli* as the most common Gram-negative isolates [48]. As in our study, *P. aeruginosa* demonstrated low resistance to ceftolozane-tazobactam. However, Enterobacterales in the Canadian study exhibited a lower prevalence of carbapenem resistance and a higher resistance to colistin compared to our findings. In our study, mortality was highest for *K. pneumoniae* and *A. baumannii*, particularly among ICU-admitted patients. Survival analysis revealed lower survival rates for *A. baumannii*, *S. maltophilia*, and *K. pneumoniae*, also during the first 14 days after blood culture collection. Adjusted multivariable analysis confirmed that only *K. pneumoniae* and *A. baumannii* isolation were independently associated with higher mortality (aOR: 1.655, 95% CI: 1.143–2.397 and aOR: 1.393, 95% CI: 1.032–1.881, respectively). Recent studies corroborate our findings, indicating shorter survival for pneumonia-related *A. baumannii* bacteremia [49] and highlighting the significant mortality risk posed by carbapenem-resistant *K. pneumoniae* and *A. baumannii* [50,51]. A recent systematic review revealed that *A. baumannii* BSIs had the highest in-hospital mortality rate (55.4%). The top three antibiotic-resistant bacterium types, cefepime-resistant *P. aeruginosa*, carbapenem-resistant *E. coli*, and carbapenem-resistant *A. baumannii*, had the highest mortality rates at 28 or 30 days, all exceeding 50% [52].

Finally, studies like ours are valuable locally for understanding epidemiological trends, guiding rational empirical antibiotic therapy selection, and informing targeted interventions through antimicrobial stewardship programs. Antimicrobial stewardship programs have been implemented in Sicily over the past four years to combat AMR [8]. In Sicily, 15,373 hospitalizations associated with sepsis occurred from 2016 to 2020, with an overall in-hospital mortality rate of 36.3%. The percentage of hospitalizations associated with sepsis represented 0.65% of all admissions, with an increase over the years [53].

An antimicrobial stewardship team, comprising infectious diseases specialists, clinical pharmacists, and microbiologists, has been established at our University Policlinic Hospital. Specific antibiotic treatment protocols have been developed based on the type of infection, and the infectious diseases specialist is routinely consulted to validate or adjust antimicrobial therapy in all cases of hospital-acquired infections. With a more rigorous control over antibiotic prescriptions, we hope to curb the levels of antimicrobial resistance in the coming years. Moreover, contact isolation remains a key infection control measure, particularly in cases involving MDR pathogens such as MBL-producing Gram-negative bacteria, which are increasingly prevalent in our geographical area.

## 4. Limits

Our study is single-center; therefore, the results cannot be generalized to the entire Sicilian or Italian territory. A significant limitation is the lack of detailed clinical data, aside from the information from hospital discharge records, which does not allow for a direct correlation between in-hospital mortality and the identified BSIs. Based on our data, we could not distinguish contaminants from true BSIs, particularly in the case of CoNS. Furthermore, the temporal relationship between BSIs and other associated infections could not be established, as it remains unclear whether these infections preceded, occurred concurrently with, or followed the BSI. An additional limitation is the absence of data on antimicrobial therapy, which prevents evaluating its impact on patient outcomes. Similarly, we cannot ascertain whether mortality was directly attributable to the isolated microorganism or other confounding factors. These limitations may lead to an overestimation of BSI-related mortality.

## 5. Materials and Methods

### 5.1. Data Collection

We conducted a single-center, retrospective, observational study. Microbiological data from all blood cultures obtained from peripheral veins and central venous catheters at the University Hospital Paolo Giaccone in Palermo between 1 January 2018 and 31 December 2024 were retrieved from an institutional anonymized electronic microbiological information system. A total of 6345 isolates from 2967 patients were collected. Patient records included age, sex, comorbidities, bacterial isolates, antimicrobial susceptibility patterns, hospital ward, LOS, and in-hospital mortality. Comorbidities were extracted from hospital discharge records, and the CCI was calculated. Univariate analysis of in-hospital mortality was performed, considering sex, LOS, ICU admission, comorbidities, infections associated with bacteremia, HIV status, and the number of isolates. Antibiotic resistance prevalence was determined using susceptibility test results interpreted according to the European Committee on Antimicrobial Susceptibility Testing (EUCAST) criteria relevant to each year of the study period [54]. Methicillin resistance in *S. aureus* isolates was determined using the Minimum Inhibitory Concentration (MIC) test (MIC values > 2 mg/L for oxacillin). Phenotypic resistance to meropenem and/or imipenem was used to assess carbapenem resistance. Additionally, cefiderocol susceptibility was tested in a subset of Gram-negative isolates. Data collection was facilitated through the Business Intelligence system Biwer, developed by Werfen [55], which enabled advanced data acquisition via native integration with the microbiology laboratory’s information system. Multiple isolates per patient were permitted; however, identical microbial isolates collected within seven days were considered duplicates and were excluded a priori. Furthermore, for statistical analyses and prevalence assessments, other duplicate isolates, defined as identical isolates from the same patient without considering the temporal criterion, were removed, and the number of unique isolates was included as a variable. We reported the annual prevalence of pathogens, the number of isolates per 1000 admitted patients, the number of isolates per 10,000 patient-days, the number of isolates per 10,000 blood cultures, and trends in antimicrobial resistance over time. Cumulative antibiograms for the main microorganism were evaluated. The cumulative antibiogram is an annual report that monitors antimicrobial resistance trends in healthcare facilities, providing a yearly summary of susceptibility rates to antibiotic therapies [56]. In 2022, the Clinical and Laboratory Standards Institute (CLSI) published a new guideline, M39—Analysis and Presentation of Cumulative Antimicrobial Susceptibility Test Data, recognizing the need to develop practical yet clinically and epidemiologically useful recommendations for analyzing and presenting data on antimicrobial susceptibility trends [57]. In this study, we evaluated the cumulative antibiograms of all the years considered in the retrospective study. Hospital wards were classified as medical, surgical, ICUs, and neonatal ICUs, with isolates categorized accordingly. Survival analyses were conducted based on isolate type for overall LOS and at 14 days from the collection of the positive blood culture. Staphylococci other than *S. aureus* were excluded from the mortality analysis, as they are typically considered contaminants. Finally, a multivariable analysis was used to assess the association between in-hospital mortality and isolated microorganisms with a lower survival rate.

### 5.2. Blood Cultures

Microorganisms isolated from positive blood cultures were identified using matrix-assisted laser desorption/ionization time-of-flight mass spectrometry (MALDI-TOF MS) with the MALDI Biotyper system (Bruker Daltonics, Billerica, MA, USA), according to the manufacturer’s instructions. Antimicrobial susceptibility testing (AST) for all antibiotics, with the exception of cefiderocol, was performed using the automated Phoenix system (Becton Dickinson Diagnostics, Sparks, MD, USA). AST results were interpreted based on the clinical breakpoints defined by the European Committee on Antimicrobial Susceptibility Testing (EUCAST), with the version of the guidelines corresponding to the respective year of testing. Cefiderocol susceptibility testing was carried out using a gradient diffusion method (Liofilchem S.r.l., Roseto degli Abruzzi, Italy) with a concentration range of 0.016–256 mg/L. Minimum inhibitory concentrations (MICs) were interpreted in accordance with the applicable EUCAST breakpoints and recommendations at the time of testing.

### 5.3. Statistical Analysis

Categorical variables are presented as numbers and percentages, while continuous variables are expressed as medians and interquartile ranges (IQRs). Differences in medians were assessed using the Mann–Whitney U test, while categorical variables were analyzed using the chi-square (χ^2^) test. Analysis of variance (ANOVA) was performed to evaluate differences across three or more groups. Pearson correlation was used to measure the linear correlation between bacterial species, specific infections diagnosed during hospitalization, and the relationship between bacterial species and wards. Annual trends in the percentage of microbiological isolates and AMR were investigated using a linear regression model with estimates of unstandardized coefficients (ß) and their confidence intervals (95% CI). Statistical significance was defined as a *p*-value of less than 0.05. Crude odds ratios (cORs) and their 95% confidence intervals (CIs) were calculated using univariate analysis to assess the association between mortality and potential risk factors. Adjusted odds ratios (aORs) were determined through logistic regression analysis to identify factors independently associated with in-hospital mortality. The logistic regression model included age, factors associated with mortality in univariate analysis, and isolates linked to lower survival rates, as determined by Kaplan–Meier curves. All statistical analyses were conducted using IBM SPSS, version 26, and StatPlus: Mac.

## 6. Conclusions

This extensive, multi-year surveillance study highlights the substantial burden of BSIs in hospitalized patients, characterized by high in-hospital mortality, prolonged length of stay, and significant antimicrobial resistance. BSIs were most prevalent in medical wards but were associated with the highest mortality rates in ICUs, particularly when caused by *Acinetobacter baumannii* and *Klebsiella pneumoniae*. These pathogens were also linked to high resistance to multiple antibiotics, including last-resort agents. Despite the overall stable incidence of BSIs per hospital admission, the proportion of deaths involving bacteremia has increased over time, suggesting a growing clinical impact. The emergence and persistence of multidrug-resistant organisms, especially carbapenem-resistant *K. pneumoniae*, multidrug-resistant *A. baumannii*, and vancomycin-resistant enterococci, further underscore the urgency of strengthening infection prevention, early diagnostic strategies, and antimicrobial stewardship. Our findings provide valuable insight into the evolving epidemiology of BSIs and their outcomes, informing clinical decision making and public health strategies aimed at reducing BSI-related morbidity and mortality.

## Figures and Tables

**Table 1:** In-hospital mortality in univariate analysis by sex, ward, LOS, comorbidities, and associated infections.

Variable	Alive at Discharge N = 2168	Dead at Discharge N = 799	cOR	*p*
Male	60.56% (1313)	54.82% (438)	0.79	0.005
ICU	3.69% (80)	55.19% (441)	32.15	<0.001
LOS (mean ± SD)	30.81 ± 26.13	31.98 ± 31.13		0.308
CCI	3.38 ± 2.12	3.54 ± 1.72		0.048
Septic shock	3.74% (81)	30.41% (243)	11.26	<0.001
Pneumonia	22.69% (492)	33.04% (256)	1.61	<0.001
Endocarditis	5.67% (123)	3.25% (26)	0.56	0.007
UTI	12.64% (274)	3.63% (29)	0.26	<0.001
IAI	6.78% (147)	2.50% (20)	0.35	<0.001
CNSI	1.15% (25)	2.38% (19)	2.09	0.014
OI	2.35% (51)	0.50% (4)	0.21	<0.001
SSTI	0.64% (14)	0.62% (5)		0.952
COVID-19	3.04% (66)	5.26% (42)	1.77	0.004
HIV	1.89% (41)	1.13% (9)		0.151
Number of isolates	1.90 ± 1.91	2.79 ± 3.21		<0.001

ICU: intensive care unit; LOS: length of stay; CCI: Charlson Comorbidity Index; UTI: urinary tract infection; IAI: intra-abdominal infection; CNSI: central nervous system infection; OI: osteoarticular infection; SSTI: skin and soft tissue infection.

**Table 2:** Prevalence of all isolates by hospital ward.

Bacteria	Overall N = 5324	Medicine 2989	Surgery 729	ICU 1471	NICU 135
*Staphylococcus* spp. *S. epidermidis* *S. aureus* *S. haemolyticus* *S. hominis* *S. capitis* *S. pettenkoferi* *S. caprae* *S. lugdunensis* *S. warneri* *S. cohnii* *S. pasteuri* *S. simulans* *S. saprophyticus* *S. equorum* *S. condimenti* *S. intermedius* *S. lentus* *S. xylosus* *S. sciuri* *S. kloosi* Other CONS Other COPS Others	2510 (47.1%) 1063 (20.0%) 398 (7.5%) 416 (7.8%) 339 (6.4%) 125 (2.3%) 52 (1.0%) 24 (0.4%) 12 (0.2%) 13 (0.2%) 14 (0.2%) 4 (0.1%) 4 (0.1%) 3 (<0.1%) 2 (<0.1%) 1 (<0.1%) 1 (<0.1%) 1 (<0.1%) 1 (<0.1%) 1 (<0.1%) 1 (<0.1%) 13 (0.2%) 2 (<0.1%) 20 (0.4%)	1499 (50.1%) 595 (19.9%) 281 (9.4%) 240 (8.0%) 214 (7.1%) 75 (2.5%) 28 (0.9%) 15 (0.5%) 9 (0.3%) 6 (0.2%) 8 (0.3%) 1 (<0.1%) 4 (0.1%) 1 (<0.1%) 1 (<0.1%) 1 (<0.1%) 1 (<0.1%) 1 (<0.1%) 1 (<0.1%) 0 (0.0%) 0 (0.0%) 6 (0.2%) 1 (<0.1%) 10 (0.3%)	347 (47.6%) 151 (20.7%) 55 (7.5%) 63 (8.6%) 51 (7.0%) 9 (1.2%) 6 (0.8%) 0 (0.0%) 1 (0.1%) 1 (0.1%) 2 (0.3%) 1 (0.1%) 0 (0.0%) 0 (0.0%) 1 (0.1%) 0 (0.0%) 0 (0.0%) 0 (0.0%) 0 (0.0%) 0 (0.0%) 1 (0.1%) 2 (0.3%) 0 (0.0%) 3 (0.4%)	572 (38.9%) 264 (17.9%) 57 (3.9%) 97 (6.6%) 66 (4.5%) 39 (2.6%) 18 (1.2%) 9 (0.6%) 2 (0.1%) 2 (0.1%) 4 (0.3%) 1 (0.1%) 0 (0.0%) 1 (0.1%) 0 (0.0%) 0 (0.0%) 0 (0.0%) 0 (0.0%) 0 (0.0%) 1 (0.1%) 0 (0.0%) 5 (0.3%) 0 (0.0%) 6 (0.4%)	92 (68.1%) 53 (39.2%) 5 (3.7%) 16 (11.8%) 8 (5.9%) 2 (1.5%) 0 (0.0%) 0 (0.0%) 0 (0.0%) 4 (3.0%) 0 (0.0%) 1 (0.7%) 0 (0.0%) 1 (0.7%) 0 (0.0%) 0 (0.0%) 0 (0.0%) 0 (0.0%) 0 (0.0%) 0 (0.0%) 0 (0.0%) 0 (0.0%) 1 (0.7%) 1 (0.7%)
*Enterococcus* spp. *E. faecalis* *E. faecium* *E. avium* *E. casseliflavus* *E. durans* *E. gallinarum* *E. hirae* *E. raffinosus*	440 (8.3%) 234 (4.4%) 193 (3.6%) 3 (<0.1%) 3 (<0.1%) 1 (<0.1%) 3 (<0.1%) 1 (<0.1%) 2 (<0.1%)	237 (7.9%) 115 (3.8%) 112 (3.7%) 3 (0.1%) 2 (0.1%) 1 (<0.1%) 3 (0.1%) 0 (0.0%) 1 (<0.1%)	72 (9.9%) 43 (5.9%) 28 (3.8%) 0 (0.0%) 0 (0.0%) 0 (0.0%) 0 (0.0%) 1 (0.1%) 0 (0.0%)	121 (8.2%) 69 (4.7%) 50 (3.4%) 0 (0.0%) 1 (0.1%) 0 (0.0%) 0 (0.0%) 0 (0.0%) 1 (0.1%)	10 (7.4%) 7 (5.2%) 3 (2.2%) 0 (0.0%) 0 (0.0%) 0 (0.0%) 0 (0.0%) 0 (0.0%) 0 (0.0%)
*E. coli*	380 (7.1%)	289 (9.7%)	50 (6.8%)	38 (2.6%)	3 (2.2%)
*Pseudomonas* spp. *P. aeruginosa* *P. oryzihabitans* *P. putida* *P. stutzeri* Others	234 (4.4%) 224 (4.2%) 1 (<0.1%) 1 (<0.1%) 1 (<0.1%) 7 (0.1%)	84 (2.8%) 81 (2.7%) 1 (<0.1%) 1 (<0.1%) 1 (<0.1%) 0 (0.0%)	31 (4.2%) 30 (4.1%) 0 (0.0%) 0 (0.0%) 0 (0.0%) 1 (0.1%)	117 (7.9%) 111 (7.5%) 0 (0.0%) 0 (0.0%) 0 (0.0%) 6 (0.4%)	2 (1.5%) 2 (1.5%) 0 (0.0%) 0 (0.0%) 0 (0.0%) 0 (0.0%)
*Klebsiella* spp. *K. pneumoniae* *K. oxytoca* *K. ozaenae* Others	693 (13.0%) 646 (12.1%) 29 (0.5%) 4 (0.1%) 13 (0.2%)	344 (11.5%) 311 (10.4%) 23 (0.8%) 2 (0.1%) 8 (0.3%)	98 (13.4%) 91 (12.5%) 4 (0.5%) 2 (0.3%) 1 (0.1%)	241 (16.4%) 235 (16.0%) 2 (0.1%) 0 (0.0%) 4 (0.3%)	10 (7.4%) 9 (6.7%) 0 (0.0%) 0 (0.0%) 1 (0.7%)
*Enterobacter* spp. *E. cloacae* *E. aerogenes* *E. hormaechei* *E. kobei* *E. asburiae* *E. gergoviae* *E. sakazakii* Others	122 (2.3%) 75 (1.4%) 22 (0.4%) 15 (0.3%) 2 (<0.1%) 2 (<0.1%) 1 (<0.1%) 1 (<0.1%) 4 (0.1%)	64 (2.1%) 39 (1.3%) 13 (0.4%) 6 (0.2%) 0 (0.0%) 1 (<0.1%) 1 (<0.1%) 1 (<0.1%) 3 (0.1%)	22 (3.0%) 17 (2.3%) 3 (0.4%) 2 (0.3%) 0 (0.0%) 0 (0.0%) 0 (0.0%) 0 (0.0%) 0 (0.0%)	30 (2.0%) 14 (0.9%) 6 (0.4%) 6 (0.4%) 1 (0.1%) 1 (0.1%) 0 (0.0%) 1 (0.1%) 1 (0.1%)	6 (4.4%) 5 (3.7%) 0 (0.0%) 1 (0.7%) 0 (0.0%) 0 (0.0%) 0 (0.0%) 0 (0.0%) 0 (0.0%)
*Citrobacter* spp. *C. koseri* *C. freundii* *C. farmeri* *C. amalonaticus*	10 (0.2%) 5 (0.1%) 3 (<0.1%) 1 (<0.1%) 1 (<0.1%)	6 (0.2%) 4 (0.1%) 2 (0.1%) 0 (0.0%) 0 (0.0%)	2 (0.3%) 0 (0.0%) 0 (0.0%) 1 (0.1%) 1 (0.1%)	2 (0.1%) 1 (0.1%) 1 (0.1%) 0 (0.0%) 0 (0.0%)	0 (0.0%) 0 (0.0%) 0 (0.0%) 0 (0.0%) 0 (0.0%)
*Morganella morgannii*	6 (0.1%)	3 (0.1%)	0 (0.0%)	3 (0.2%)	0 (0.0%)
*Serratia* spp. *S. marcensces* *S. liquefaciens* *S. plymuthica* Others	70 (1.3%) 52 (1.1%) 4 (0.1%) 3 (<0.1%) 11 (0.2%)	17 (0.6%) 12 (0.4%) 1 (< 0.1%) 3 (0.1%) 2 (0.1%)	13 (1.8%) 12 (1.6%) 0 (0.0%) 0 (0.0%) 1 (0.1%)	39 (2.6%) 28 (1.9%) 3 (0.2%) 0 (0.0%) 8 (0.5%)	1 (0.7%) 1 (0.7%) 0 (0.0%) 0 (0.0%) 0 (0.0%)
*Acinetobacter* spp. *A. baumannii* *A. lwoffii* Others	415 (7.8%) 396 (7.4%) 7 (0.1%) 12 (0.2%)	165 (5.5%) 154 (5.1%) 3 (0.1%) 8 (0.3%)	35 (4.8%) 34 (4.7%) 1 (0.1%) 0 (0.0%)	214 (14.5%) 208 (14.1%) 2 (0.1%) 4 (0.3%)	1 (0.7%) 0 (0.0%) 1 (0.7%) 0 (0.0%)
*Proteus* spp. *P. mirabilis* *P. vulgaris* *P. hauseri*	57 (1.0%) 54 (1.0%) 2 (<0.1%) 1 (<0.1%)	35 (1.2%) 34 (1.1%) 0 (0.0%) 1 (<0.1%)	9 (1.2%) 8 (1.1%) 1 (0.1%) 0 (0.0%)	13 (0.9%) 12 (0.8%) 1 (0.1%) 0 (0.0%)	0 (0.0%) 0 (0.0%) 0 (0.0%) 0 (0.0%)
*Providencia* spp. *P. stuartii* *P. rustigianii*	19 (0.3%) 18 (0.3%) 1 (<0.1%)	7 (0.2%) 6 (0.2%) 1 (<0.1%)	3 (0.4%) 3 (0.4%) 0 (0.0%)	9 (0.6%) 9 (0.6%) 0 (0.0%)	0 (0.0%) 0 (0.0%) 0 (0.0%)
*Stenotrophomonas maltophilia*	44 (0.8%)	16 (0.5%)	5 (0.7%)	22 (1.5%)	1 (0.7%)
*Streptococcus* spp. *S. bovis* *S. gallolyticus* *S. anginosus* *S. pneumoniae* *S. agalactiae* *S. constellatus* *S. dysgalactiae* *S. pyogens* *S. sanguinis* *S. parasanguinis* *S. acidominimus* *S. cristatus* *S. equi* *S. gordonii* *S. salivarius* *S. intermedius* *S. mitis* *S. oralis* Others	96 (1.8%) 10 (0.2%) 8 (0.1%) 8 (0.1%) 6 (0.1%) 5 (0.1%) 4 (0.1%) 2 (<0.1%) 5 (0.1%) 3 (<0.1%) 3 (<0.1%) 2 (<0.1%) 1 (<0.1%) 1 (<0.1%) 1 (<0.1%) 1 (<0.1%) 4 (0.1%) 15 (0.3%) 9 (0.2%) 8 (0.1%)	73 (2.4%) 10 (0.3%) 6 (0.2%) 5 (0.2%) 6 (0.2%) 3 (0.1%) 3 (0.1%) 1 (<0.1%) 5 (0.2%) 0 (0.0%) 3 (0.1%) 2 (0.1%) 0 (0.0%) 1 (<0.1%) 1 (<0.1%) 0 (0.0%) 3 (0.1%) 10 (0.3%) 8 (0.3%) 6 (0.2%)	14 (1.9%) 0 (0.0%) 2 (0.3%) 2 (0.3%) 0 (0.0%) 0 (0.0%) 0 (0.0%) 1 (0.1%) 0 (0.0%) 3 (0.4%) 0 (0.0%) 0 (0.0%) 1 (0.1%) 0 (0.0%) 0 (0.0%) 0 (0.0%) 1 (0.1%) 2 (0.3%) 1 (0.1%) 1 (0.1%)	7 (0.5%) 0 (0.0%) 0 (0.0%) 1 (0.1%) 0 (0.0%) 1 (0.1%) 1 (0.1%) 0 (0.0%) 0 (0.0%) 0 (0.0%) 0 (0.0%) 0 (0.0%) 0 (0.0%) 0 (0.0%) 0 (0.0%) 1 (0.1%) 0 (0.0%) 2 (0.1%) 0 (0.0%) 1 (0.1%)	2 (1.5%) 0 (0.0%) 0 (0.0%) 0 (0.0%) 0 (0.0%) 1 (0.7%) 0 (0.0%) 0 (0.0%) 0 (0.0%) 0 (0.0%) 0 (0.0%) 0 (0.0%) 0 (0.0%) 0 (0.0%) 0 (0.0%) 0 (0.0%) 0 (0.0%) 1 (0.7%) 0 (0.0%) 0 (0.0%)
*Corynebacterium* spp. *C. striatum* *C. afermentas* *C. jeikeium* *C. amycolatum* *C. coyleae* *C. falseni* *C. imitans* *C. matruchotii* *C. mucifaciens* *C. propinquum* *C. urealyticum* Others	100 (1.9%) 71 (1.3%) 6 (0.1%) 6 (0.1%) 5 (0.1%) 1 (<0.1%) 1 (<0.1%) 1 (<0.1%) 2 (<0.1%) 1 (<0.1%) 1 (<0.1%) 1 (<0.1%) 4 (0.1%)	68 (2.3%) 50 (1.7%) 3 (0.1%) 3 (0.1%) 2 (0.1%) 1 (<0.1%) 0 (0.0%) 1 (<0.1%) 2 (0.1%) 1 (<0.1%) 1 (<0.1%) 1 (<0.1%) 3 (0.1%)	11 (1.5%) 8 (1.1%) 2 (0.3%) 0 (0.0%) 1 (0.1%) 0 (0.0%) 0 (0.0%) 0 (0.0%) 0 (0.0%) 0 (0.0%) 0 (0.0%) 0 (0.0%) 0 (0.0%)	20 (1.3%) 13 (0.9%) 1 (0.1%) 3 (0.2%) 2 (0.1%) 0 (0.0%) 0 (0.0%) 0 (0.0%) 0 (0.0%) 0 (0.0%) 0 (0.0%) 0 (0.0%) 1 (0.1%)	1 (0.7%) 0 (0.0%) 0 (0.0%) 0 (0.0%) 0 (0.0%) 0 (0.0%) 1 (0.7%) 0 (0.0%) 0 (0.0%) 0 (0.0%) 0 (0.0%) 0 (0.0%) 0 (0.0%)
*Propionibacterium* spp. *P. acnes* Others	5 (0.1%) 4 (0.1%) 1 (<0.1%)	2 (0.1%) 2 (0.1%) 0 (0.0%)	1 (0.1%) 0 (0.0%) 1 (0.1%)	2 (0.1%) 2 (0.1%) 0 (0.0%)	0 (0.0%) 0 (0.0%) 0 (0.0%)
*Haemophilus* spp *H. parainfluenzae* *H. influenzae*	4 (0.1%) 2 (<0.1%) 2 (<0.1%)	3 (0.1%) 2 (0.1%) 1 (<0.1%)	1 (0.1%) 0 (0.0%) 1 (0.1%)	0 (0.0%) 0 (0.0%) 0 (0.0%)	0 (0.0%) 0 (0.0%) 0 (0.0%)
*Listeria monocytogenes*	14 (0.3%)	8 (0.3%)	1 (0.1%)	3 (0.2%)	2 (1.5%)
*Brucella* spp.	1 (<0.1%)	1 (<0.1%)	0 (0.0%)	0 (0.0%)	0 (0.0%)
*Salmonella typhi*	3 (<0.1%)	3 (0.1%)	0 (0.0%)	0 (0.0%)	0 (0.0%)
*Salmonella* spp.	4 (0.1%)	3 (0.1%)	1 (0.1%)	0 (0.0%)	0 (0.0%)
*Vibrio* spp.	1 (<0.1%)	1 (<0.1%)	0 (0.0%)	0 (0.0%)	0 (0.0%)
*Nocardia* spp.	1 (<0.1%)	1 (<0.1%)	0 (0.0%)	0 (0.0%)	0 (0.0%)
*Achromobacter* spp. *A. xilosoxidans* Others	20 (0.4%) 14 (0.3%) 6 (0.1%)	7 (0.2%) 5 (0.2%) 2 (0.1%)	2 (0.3%) 0 (0.0%) 2 (0.3%)	11 (0.7%) 9 (0.6%) 2 (0.1%)	0 (0.0%) 0 (0.0%) 0 (0.0%)
*Aeromonas* spp. *A. caviae* *A. veronii* *A. sobria*	5 (0.1%) 2 (<0.1%) 2 (<0.1%) 1 (<0.1%)	5 (0.2%) 2 (0.1%) 2 (0.1%) 1 (<0.1%)	0 (0.0%) 0 (0.0%) 0 (0.0%) 0 (0.0%)	0 (0.0%) 0 (0.0%) 0 (0.0%) 0 (0.0%)	0 (0.0%) 0 (0.0%) 0 (0.0%) 0 (0.0%)
*Alcaligenes faecalis*	2 (<0.1%)	1 (<0.1%)	1 (0.1%)	0 (0.0%)	0 (0.0%)
*Bacillus cereus*	2 (<0.1%)	2 (0.1%)	0 (0.0%)	0 (0.0%)	0 (0.0%)
*Bacillus licheniformis*	1 (<0.1%)	1 (<0.1%)	0 (0.0%)	0 (0.0%)	0 (0.0%)
*Bacteroides* spp. *B. fragilis* *B. caccae* *B. theiyaiotamicron*	15 (0.3%) 11 (0.2%) 2 (<0.1%) 2 (<0.1%)	9 (0.3%) 6 (0.2%) 1 (<0.1%) 2 (0.1%)	4 (0.5%) 4 (0.5%) 0 (0.0%) 0 (0.0%)	2 (0.1%) 1 (0.1%) 1 (0.1%) 0 (0.0%)	0 (0.0%) 0 (0.0%) 0 (0.0%) 0 (0.0%)
*Brevibacterium* spp. *B. casei* Others	3 (<0.1%) 2 (<0.1%) 1 (<0.1%)	3 (0.1%) 2 (0.1%) 1 (<0.1%)	0 (0.0%) 0 (0.0%) 0 (0.0%)	0 (0.0%) 0 (0.0%) 0 (0.0%)	0 (0.0%) 0 (0.0%) 0 (0.0%)
*Fusobacterium* spp. *F. necrophorum* *F. nucleatum*	3 (<0.1%) 2 (<0.1%) 1 (<0.1%)	0 (0.0%) 0 (0.0%) 0 (0.0%)	2 (0.3%) 2 (0.3%) 0 (0.0%)	1 (0.1%) 0 (0.0%) 1 (0.1%)	0 (0.0%) 0 (0.0%) 0 (0.0%)
*Clostridioides* spp. *C. paraputrificum* *C. perfringens* *C. septicum* *C. sporogenes*	6 (0.1%) 2 (<0.1%) 2 (<0.1%) 1 (<0.1%) 1 (<0.1%)	5 (0.2%) 2 (0.1%) 1 (<0.1%) 1 (<0.1%) 1 (<0.1%)	1 (0.1%) 0 (0.0%) 1 (0.1%) 0 (0.0%) 0 (0.0%)	0 (0.0%) 0 (0.0%) 0 (0.0%) 0 (0.0%) 0 (0.0%)	0 (0.0%) 0 (0.0%) 0 (0.0%) 0 (0.0%) 0 (0.0%)
*Brevundimonas vesicularis*	1 (<0.1%)	1 (<0.1%)	0 (0.0%)	0 (0.0%)	0 (0.0%)
*Burkholderia* spp.	1 (<0.1%)	1 (<0.1%)	0 (0.0%)	0 (0.0%)	0 (0.0%)
*Cedecea davisae*	1 (<0.1%)	0 (0.0%)	0 (0.0%)	1 (0.1%)	0 (0.0%)
*Delftia acidovorans*	1 (<0.1%)	1 (<0.1%)	0 (0.0%)	0 (0.0%)	0 (0.0%)
*Dermabacter hominis*	1 (<0.1%)	1 (<0.1%)	0 (0.0%)	0 (0.0%)	0 (0.0%)
*Escherichia hermannii*	1 (<0.1%)	1 (<0.1%)	0 (0.0%)	0 (0.0%)	0 (0.0%)
*Gemella haemolysans*	1 (<0.1%)	1 (<0.1%)	0 (0.0%)	0 (0.0%)	0 (0.0%)
*Hafnia alvei*	4 (0.1%)	2 (0.1%)	0 (0.0%)	2 (0.1%)	0 (0.0%)
*Lactobacillus rhamnosus*	2 (<0.1%)	0 (0.0%)	1 (0.1%)	1 (0.1%)	0 (0.0%)
*Lactococcus garvieae*	1 (<0.1%)	0 (0.0%)	0 (0.0%)	0 (0.0%)	1 (0.7%)
*Leclercia adecarboxylata*	1 (<0.1%)	1 (<0.1%)	0 (0.0%)	0 (0.0%)	0 (0.0%)
*Micrococcus luteus*	1 (<0.1%)	1 (<0.1%)	0 (0.0%)	0 (0.0%)	0 (0.0%)
*Moraxella* spp. *M. osloensis* *M. atlantae*	3 (<0.1%) 2 (<0.1%) 1 (<0.1%)	3 (0.1%) 2 (0.1%) 1 (<0.1%)	0 (0.0%) 0 (0.0%) 0 (0.0%)	0 (0.0%) 0 (0.0%) 0 (0.0%)	0 (0.0%) 0 (0.0%) 0 (0.0%)
*Neisseria flavescens*	1 (<0.1%)	1 (<0.1%)	0 (0.0%)	0 (0.0%)	0 (0.0%)
*Ochrobactrum anthropii*	2 (<0.1%)	1 (<0.1%)	1 (0.1%)	0 (0.0%)	0 (0.0%)
*Oligella ureolytica*	1 (<0.1%)	1 (<0.1%)	0 (0.0%)	0 (0.0%)	0 (0.0%)
*Pantoea agglomerans*	2 (<0.1%)	1 (<0.1%)	1 (0.1%)	0 (0.0%)	0 (0.0%)
*Raoultella ornithinolytica*	1 (<0.1%)	1 (<0.1%)	0 (0.0%)	0 (0.0%)	0 (0.0%)
*Rodococcus equi*	1 (<0.1%)	1 (<0.1%)	0 (0.0%)	0 (0.0%)	0 (0.0%)
*Roseomonas* spp.	3 (<0.1%)	3(0.1%)	0 (0.0%)	0 (0.0%)	0 (0.0%)
*Rothia* spp. *R. mucilaginosa* Others	2 (<0.1%) 1 (<0.1%) 1 (<0.1%)	0 (0.0%) 0 (0.0%) 0 (0.0%)	0 (0.0%) 0 (0.0%) 0 (0.0%)	0 (0.0%) 0 (0.0%) 0 (0.0%)	2 (1.5%) 1 (0.7%) 1 (0.7%)
*Stomatococcus* spp.	1 (<0.1%)	0 (0.0%)	0 (0.0%)	0 (0.0%)	1 (0.7%)
*Tissierella praeacuta*	1 (<0.1%)	1 (<0.1%)	0 (0.0%)	0 (0.0%)	0 (0.0%)
Non-identified	3 (<0.1%)	3 (0.1%)	0 (0.0%)	0 (0.0%)	0 (0.0%)

**Table 3:** Antimicrobial resistance of major blood culture isolates from 2018 to 2024.

Bacteria	N = 5324	2018 N = 501	2019 N = 580	2020 N = 733	2021 N = 624	2022 N = 819	2023 N = 1078	2024 N = 989
Staphylococci excluding *S. aureus*	2113	198	221	322	283	352	404	333
Oxacillin	1716/2100 (81.7%)	158/196 (80.6%)	179/221 (81.0%)	260/319 (81.5%)	232/283 (82.0%)	289/352 (82.1%)	331/398 (83.2%)	267/331 (80.7%)
Ciprofloxacin	1498/2097 (71.4%)	148/198 (74.7%)	157/219 (71.7%)	230/320 (71.9%)	202/283 (71.4%)	246/352 (69.9%)	295/399 (73.9%)	220/326 (67.5%)
Moxifloxacin	1428/2054 (69.5%)	126/184 (68.5%)	147/210 (70.0%)	225/318 (70.7%)	198/282 (70.2%)	244/350 (69.7%)	282/392 (71.9%)	206/318 (64.8%)
TMP-SMX	805/2088 (38.5%)	60/196 (30.6%)	81/219 (37.0%)	117/318 (36.8%)	133/282 (47.2%)	139/350 (39.7%)	158/398 (39.7%)	117/325 (36.0%)
Gentamicin	1421/2100 (67.7%)	118/197 (59.9%)	141/219 (64.4%)	216/320 (67.5%)	206/283 (72.8%)	249/352 (70.7%)	269/398 (67.6%)	222/331 (67.0%)
Tetracycline	472/2090 (22.6%)	48/196 (24.5%)	52/219 (23.7%)	90/318 (28.3%)	78/281 (27.7%)	57/348 (16.4%)	66/398 (16.6%)	81/330 (24.5%)
Tigecycline	108/2062 (5.2%)	18/184 (9.8%)	7/208 (3.4%)	17/316 (5.4%)	16/280 (5.7%)	20/350 (5.7%)	6/398 (1.5%)	24/326 (7.4%)
Clindamycin	1280/2082 (61.5%)	105/193 (54.4%)	119/211 (56.4%)	181/317 (57.1%)	181/281 (64.4%)	217/351 (61.8%)	262/398 (65.8%)	215/331 (64.9%)
Erythromycin	1652/2101 (78.6%)	141/196 (71.9%)	168/221 (76.0%)	245/320 (76.6%)	235/283 (83.0%)	288/352 (81.8%)	326/398 (81.9%)	249/331 (75.2%)
Teicoplanin	242/2046 (11.8%)	4/191 (2.1%)	19/215 (8.8%)	33/313 (10.5%)	47/280 (16.8%)	71/328 (21.6%)	33/394 (8.4%)	35/325 (10.8%)
Vancomycin	12/2110 (0.6%)	1/198 (0.5%)	1/221 (0.4%)	3/321 (0.9%)	2/283 (0.7%)	1/352 (0.3%)	4/402 (1.0%)	0/333 (0.0%)
Linezolid	343/2100 (16.3%)	26/195 (13.3%)	34/214 (15.9%)	39/319 (12.2%)	48/283 (17.0%)	66/352 (18.7%)	70/404 (17.3%)	60/333 (18.0%)
Daptomycin	26/2084 (1.2%)	2/186 (1.1%)	3/210 (1.4%)	2/319 (0.6%)	5/283 (1.8%)	2/352 (0.6%)	7/401 (1.7%)	5/333 (1.5%)
Rifampicin	849/920 (92.3%)	52/115 (45.2%)	65/68 (95.6%)	116/118 (98.3%)	128/128 (100%)	162/163 (99.4%)	179/180 (99.4%)	147/148 (99.3%)
Fosfomycin	1105/2075 (53.2%)	52/186 (27.9%)	88/209 (42.1%)	144/318 (45.3%)	176/283 (62.2%)	195/351 (55.5%)	251/398 (63.1%)	199/330 (60.3%)
*K. pneumoniae*	646	66	73	89	75	74	123	146
TZP	516/645 (80.0%)	55/66 (83.3%)	57/73 (78.1%)	70/89 (78.6%)	66/75 (88.0%)	60/74 (81.1%)	89/123 (72.3%)	119/145 (82.1%)
Cefepime	542/644 (84.2%)	55/66 (83.3%)	60/73 (82.2%)	77/89 (86.5%)	67/75 (89.3%)	62/72 (86.1%)	98/123 (79.7%)	123/146 (84.2%)
Ceftazidime	557/646 (86.2%)	56/66 (83.3%)	61/73 (83.6%)	77/89 (86.5%)	68/75 (90.7%)	65/74 (87.8%)	105/123 (85.4%)	125/146 (85.6%)
CZA	58/491 (11.8%)	NT	0/26 (0.0%)	3/52 (5.8%)	12/74 (16.2%)	3/72 (4.2%)	18/123 (14.6%)	22/144 (15.3%)
CZT	313/432 (72.4%)	NT	NT	16/22 (72.7%)	65/74 (87.8%)	51/68 (75.0%)	78/122 (63.9%)	103/146 (70.5%)
Ertapenem	470/644 (73.0%)	49/66 (74.2%)	52/73 (71.2%)	68/88 (77.3%)	65/75 (86.7%)	55/74 (74.3%)	79/123 (64.2%)	102/145 (70.3%)
Imipenem	415/641 (64.7%)	36/65 (55.4%)	39/73 (53.4%)	60/87 (69.0%)	61/75 (81.3%)	53/74 (71.6%)	73/123 (59.3%)	93/144 (64.6%)
Meropenem	404/642 (62.9%)	36/66 (54.5%)	41/73 (56.2%)	57/88 (64.8%)	60/75 (80.0%)	52/72 (72.2%)	74/123 (60.2%)	84/145 (57.9%)
MEV	41/362 (11.3%)	NT	NT	NT	0/31 (0.0%)	0/66 (0.0%)	21/122 (17.2%)	20/143 (14.0%)
Gentamicin	401/646 (62.1%)	39/66 (59.1%)	36/73 (49.3%)	64/89 (71.9%)	57/75 (76.0%)	47/74 (63.5%)	59/123 (48.0%)	99/146 (67.8%)
Ciprofloxacin	536/646 (83.0%)	54/66 (81.8%)	59/73 (80.8%)	77/89 (86.5%)	66/75 (88.0%)	65/74 (87.8%)	93/123 (75.6%)	122/146 (83.6%)
TMP-SMX	354/645 (54.9%)	35/66 (53.0%)	33/73 (45.2%)	50/88 (56.8%)	40/75 (53.3%)	33/74 (44.6%)	64/123 (52.0%)	99/146 (67.8%)
Fosfomycin	188/644 (29.2%)	19/66 (28.8%)	23/73 (31.5%)	12/88 (13.6%)	17/75 (22.7%)	26/74 (35.1%)	49/123 (39.8%)	42/145 (29.0%)
Colistin	45/626 (7.2%)	3/62 (4.8%)	4/72 (5.5%)	14/85 (16.5%)	11/73 (15.1%)	4/71 (5.6%)	8/121 (6.6%)	1/142 (0.7%)
*S. aureus*	398	38	62	49	38	60	71	80
Oxacillin	117/398 (29.4%)	16/38 (42.1%)	20/62 (32.2%)	24/49 (49.0%)	9/38 (23.7%)	11/60 (18.3%)	14/71 (19.7%)	23/80 (28.7%)
Ciprofloxacin	108/396 (27.3%)	18/38 (47.4%)	21/62 (33.9%)	16/48 (33.3%)	10/38 (26.3%)	10/60 (16.7%)	15/70 (21.4%)	18/80 (22.5%)
Moxifloxacin	103/386 (26.7%)	16/35 (45.7%)	21/62 (33.9%)	16/48 (33.3%)	10/36 (27.8%)	10/60 (16.7%)	13/67 (19.4%)	17/78 (21.8%)
TMP-SMX	19/395 (4.8%)	5/37 (13.5%)	5/62 (8.0%)	1/48 (2.1%)	4/38 (10.5%)	0/59 (0.0%)	1/71 (1.4%)	3/80 (3.7%)
Gentamicin	61/396 (15.4%)	8/38 (21.0%)	14/62 (22.6%)	7/48 (14.6%)	7/38 (18.4%)	6/60 (10.0%)	9/70 (12.9%)	10/80 (12.5%)
Tetracycline	46/396 (11.6%)	4/38 (10.5%)	7/62 (11.3%)	11/48 (22.9%)	6/38 (15.8%)	5/60 (8.3%)	8/70 (11.4%)	5/80 (6.2%)
Tigecycline	5/392 (1.3%)	2/35 (5.7%)	1/61 (1.6%)	1/48 (2.0%)	0 (0.0%)	0 (0.0%)	1/70 (1.4%)	NT
Clindamycin	137/395 (34.7%)	11/37 (29.7%)	26/62 (41.9%)	15/48 (31.2%)	14/38 (36.8%)	24/60 (40.0%)	18/70 (25.7%)	29/80 (36.2%)
Erythromycin	146/396 (36.9%)	14/38 (36.8%)	26/62 (41.9%)	19/48 (39.6%)	15/38 (39.5%)	23/60 (38.3%)	19/70 (27.1%)	30/80 (37.5%)
Teicoplanin	0/396 (0.0%)	0/38 (0.0%)	0/62 (0.0%)	0/48 (0.0%)	0/38 (0.0%)	0/60 (0.0%)	0/70 (0.0%)	0/80 (0.0%)
Vancomycin	3/398 (0.7%)	0/38 (0.0%)	2/62 (3.2%)	0/49 (0.0%)	0/38 (0.0%)	0/60 (0.0%)	1/71 (1.4%)	0/80 (0.0%)
Linezolid	1/397 (0.2%)	0/37 (0.0%)	0/62 (0.0%)	0/49 (0.0%)	0/38 (0.0%)	0/60 (0.0%)	0/71 (0.0%)	1/80 (1.2%)
Daptomycin	6/396 (1.5%)	0/98 (0.0%)	0/62 (0.0%)	1/49 (2.0%)	2/38 (5.3%)	1/60 (1.7%)	2/71 (2.8%)	0/80 (0.0%)
Rifampicin	29/43 (67.4%)	5/18 (27.8%)	6/7 (85.7%)	2/2 (100%)	4/4 (100%)	1/1 (100%)	4/4 (100%)	7/7 (100%)
Fosfomycin	27/394 (6.8%)	5/36 (13.9%)	4/62 (6.4%)	3/48 (6.2%)	3/38 (7.9%)	4/60 (6.7%)	5/70 (7.1%)	3/80 (3.7%)
*A. baumannii*	396	24	42	49	38	67	90	86
Meropenem	383/395 (97.0%)	23/24 (95.8%)	38/42 (90.5%)	44/48 (91.7%)	37/38 (97.4%)	67/67 (100%)	90/90 (100%)	84/86 (97.7%)
Gentamicin	349/393 (88.8%)	23/24 (95.8%)	38/42 (90.5%)	43/46 (93.5%)	37/38 (97.4%)	53/67 (79.1%)	73/89 (82.0%)	82/86 (95.3%)
Ciprofloxacin	379/394 (96.2%)	24/24 (100%)	39/40 (97.5%)	45/46 (97.8%)	37/38 (97.4%)	62/62 (100%)	88/88 (100%)	84/84 (100%)
TMP-SMX	347/395 (87.8%)	22/24 (91.7%)	37/42 (88.1%)	35/48 (72.9%)	30/38 (78.9%)	60/67 (89.5%)	84/90 (93.3%)	79/86 (91.9%)
Colistin	8/391 (2.0%)	0/24 (0.0%)	3/42 (7.1%)	0/46 (0.0%)	3/38 (7.9%)	0/66 (0.0%)	1/89 (1.1%)	1/86 (1.2%)
*E. coli*	380	42	59	55	31	37	74	82
TZP	69/380 (18.1%)	5/42 (11.9%)	10/59 (16.9%)	4/55 (7.3%)	4/31 (12.9%)	8/37 (21.6%)	21/74 (28.4%)	17/82 (20.7%)
Cefepime	145/379 (38.2%)	11/42 (26.2%)	23/59 (39.0%)	24/55 (43.6%)	11/31 (35.5%)	12/37 (32.4%)	29/74 (39.2%)	35/81 (43.2%)
Ceftazidime	142/379 (37.5%)	10/42 (23.8%)	20/58 (34.5%)	22/55 (40.0%)	9/31 (29.0%)	14/37 (37.8%)	31/74 (41.9%)	36/82 (43.9%)
CZA	5/225 (2.2%)	NT	NT	0/10 (0.0%)	0/30 (0.0%)	1/31 (3.2%)	2/73 (2.7%)	2/81 (2.5%)
CZT	26/221 (11.8%)	NT	NT	0/6 (0.0%)	2/30 (6.7%)	3/31 (9.7%)	9/73 (12.3%)	12/81 (14.8%)
Ertapenem	24/378 (6.3%)	2/42 (4.8%)	2/59 (3.4%)	0/55 (0.0%)	1/31 (3.2%)	4/37 (10.8%)	8/74 (10.8%)	7/80 (8.7%)
Imipenem	8/376 (2.1%)	2/42 (4.8%)	0/59 (0.0%)	0/55 (0.0%)	0/30 (0.0%)	1/37 (2.7%)	4/74 (5.4%)	1/82 (1.2%)
Meropenem	6/380 (1.6%)	2/42 (4.8%)	0/59 (0.0%)	0/55 (0.0%)	1/31 (3.2%)	1/36 (2.8%)	1/74 (1.3%)	1/81 (1.2%)
MVB	2/127 (1.6%)	NT	NT	NT	0/1 (0%)	1/17 (5.9%)	1/55 (1.8%)	0/54 (0.0%)
Gentamicin	81/380 (21.3%)	4/42 (9.5%)	11/59 (18.6%)	19/55 (34.5%)	8/31 (25.8%)	8/37 (21.6%)	13/74 (17.6%)	18/82 (21.9%)
Ciprofloxacin	216/380 (56.8%)	26/42 (61.9%)	37/59 (62.7%)	32/55 (58.2%)	15/31 (48.4%)	17/37 (45.9%)	39/74 (52.7%)	50/82 (61.0%)
TMP-SMX	174/376 (46.3%)	19/42 (45.2%)	30/59 (50.8%)	25/54 (46.3%)	14/31 (45.2%)	16/37 (43.2%)	37/73 (50.7%)	33/80 (41.2%)
Fosfomycin	2/376 (0.5%)	1/42 (2.4%)	0/59 (0.0%)	0/52 (0.0%)	0/30 (0.0%)	0/37 (0.0%)	1/74 (1.3%)	0/82 (0.0%)
Colistin	5/348 (1.4%)	4/38 (10.5%)	0/54 (0.0%)	0/51 (0.0%)	1/28 (3.6%)	0/34 (0.0%)	0/65 (0.0%)	0/78 (0.0%)
Tigecycline	65/281 (23.1%)	0/42 (0.0%)	0/59 (0.0%)	10/48 (20.8%)	0/1 (0.0%)	4/24 (16.7%)	25/52 (48.1%)	26/55 (47.3%)
*E. faecalis*	234	24	16	37	37	37	46	37
Ampicillin	0/86 (0.0%)	0/1 (0.0%)	NT	0/1 (0.0%)	NT	0/3 (0.0%)	0/46 (0.0%)	0/37 (0.0%)
Imipenem	23/234 (9.8%)	0/24 (0.0%)	0/16 (0.0%)	3/34 (8.8%)	11/26 (42.3%)	6/31 (19.3%)	3/43 (7.0%)	0/37 (0.0%)
Gentamicin	112/234 (47.9%)	15/24 (62.5%)	9/16 (56.2%)	19/37 (51.3%)	18/37 (48.6%)	18/37 (48.6%)	21/46 (45.6%)	12/37 (32.4%)
Teicoplanin	0/234 (0.0%)	(0.0%)	(0.0%)	(0.0%)	(0.0%)	(0.0%)	(0.0%)	(0.0%)
Vancomycin	0/233 (0%)	(0.0%)	(0.0%)	(0.0%)	(0.0%)	(0.0%)	(0.0%)	(0.0%)
Linezolid	0/234 (0%)	(0.0%)	(0.0%)	(0.0%)	(0.0%)	(0.0%)	(0.0%)	(0.0%)
Tigecycline	1/233 (0.4%)	0/24 (0.0%)	0/16 (0.0%)	1/37 (2.7%)	0/37 (0.0%)	0/37 (0.0%)	0/46 (0.0%)	0/36 (0.0%)
*P. aeruginosa*	224	12	22	33	19	32	67	39
TZP	33/224 (14.7%)	5/12 (41.7%)	1/22 (4.5%)	6/33 (18.2%)	3/19 (15.8%)	2/32 (6.2%)	13/67 (19.4%)	3/39 (7.7%)
Cefepime	41/219 (18.7%)	6/12 (50.0%)	2/21 (9.5%)	8/33 (24.2%)	4/19 (21.0%)	2/32 (6.2%)	14/63 (22.2%)	5/39 (12.8%)
Ceftazidime	43/122 (35.2%)	8/12 (66.7%)	1/22 (4.5%)	6/33 (18.2%)	4/19 (21.0%)	5/32 (15.6%)	14/65 (21.5%)	5/39 (12.8%)
CZA	2/145 (1.4%)	NT	NT	0/3 (0.0%)	0/16 (0.0%)	1/31 (3.2%)	1/60 (1.7%)	0/35 (0.0%)
CTZ	3/136 (2.2%)	NT	NT	0/3 (0.0%)	0/16 (0.0%)	0/25 (0.0%)	2/58 (3.4%)	1/34 (2.9%)
Imipenem	56/217 (25.8%)	7/12 (58.3%)	6/22 (27.3%)	10/33 (30.3%)	4/19 (21.0%)	5/32 (15.6%)	16/61 (26.2%)	8/38 (21.0%)
Meropenem	36/222 (16.2%)	7/12 (58.3%)	6/21 (28.6%)	6/33 (18.2%)	3/19 (15.8%)	5/32 (15.6%)	8/66 (12.1%)	1/39 (2.6%)
Tobramycin	26/221 (11.8%)	8/12 (66.7%)	3/22 (13.6%)	7/33 (21.2%)	0/19 (0.0%)	3/31 (9.7%)	4/67 (6.0%)	1/37 (2.7%)
Amikacin	3/106 (2.8%)	NT	0/1	NT	NT	NT	3/67	0/39
Gentamicin	16/58 (27.6%)	8/12 (66.7%)	3/22 (13.6%)	5/21 (23.8%)	NT	NT	0/2 (0%)	0/1 (0%)
Ciprofloxacin	41/223 (18.4%)	8/12 (66.7%)	4/22 (18.2%)	11/33 (33.3%)	4/19 (21.0%)	3/32 (9.4%)	8/67 (11.9%)	3/38 (7.9%)
Colistin	2/218 (0.9%)	0/12 (0.0%)	0/22 (0.0%)	0/30 (0.0%)	0/19 (0.0%)	0/32 (0.0%)	2/65 (3.1%)	0/38 (0.0%)
*E. faecium*	193	16	20	27	42	20	36	32
Ampicillin	56/67 (83.6%)	NT	NT	NT	NT	NT	32/36 (88.9%)	24/31 (77.4%)
Imipenem	171/192 (89.0%)	13/16 (81.2%)	17/20 (85.0%)	26/27 (96.3%)	37/42 (88.1%)	19/20 (95.0%)	33/36 (91.7%)	26/31 (83.9%)
Gentamicin	112/192 (58.3%)	6/16 (37.5%)	12/20 (60.0%)	15/27 (55.5%)	23/42 (54.8%)	17/20 (85.0%)	21/36 (58.3%)	18/31 (58.1%)
Teicoplanin	52/192 (27.1%)	0/16 (0.0%)	0/20 (0.0%)	4/27 (14.8%)	9/42 (21.4%)	8/20 (40.0%)	14/36 (38.9%)	17/31 (54.8%)
Vancomycin	52/193 (26.9%)	0/16 (0.0%)	1/20 (5.0%)	4/27 (14.8%)	9/42 (21.4%)	7/20 (35.0%)	14/36 (38.9%)	17/32 (53.1%)
Linezolid	6/193 (3.1%)	0/16 (0.0%)	1/20 (5.0%)	0/27 (0.0%)	0/42 (0.0%)	0/20 (0.0%)	3/36 (8.3%)	2/32 (6.2%)
Q-D	46/104 (44.2%)	NT	NT	0/2 (0.0%)	1/28 (3.6%)	0/14 (0.0%)	19/34 (55.9%)	26/26 (100%)
*Enterobacter* spp.	122	20	15	11	8	25	30	13
Cefepime	33/122 (27.0%)	5/20 (25.0%)	3/15 (20.0%)	5/11 (45.4%)	3/8 (37.5%)	5/25 (20.0%)	10/30 (33.3%)	2/13 (15.4%)
Meropenem	8/122 (6.5%)	0/20 (0.0%)	1/15 (6.7%)	2/11 (18.2%)	0/8 (0.0%)	0/25 (0.0%)	4/30 (13.3%)	1/13 (7.7%)
Gentamicin	20/122 (16.4%)	3/20 (15.0%)	2/15 (13.3%)	4/11 (36.4%)	3/8 (37.5%)	2/25 (8.0%)	5/30 (16.7%)	1/13 (7.7%)
Ciprofloxacin	27/122 (22.1%)	8/20 (40.0%)	4/15 (26.7%)	4/11 (36.4%)	2/8 (25.0%)	2/25 (8.0%)	5/30 (16.7%)	2/13 (15.4%)
TMP-SMX	27/122 (22.1%)	7/20 (35.0%)	3/15 (20.0%)	4/11 (36.4%)	3/8 (37.5%)	3/25 (12.0%)	5/30 (16.7%)	2/13 (15.4%)
Fosfomycin	15/122 (12.3%)	0/20 (0.0%)	2/15 (13.3%)	3/11 (27.3%)	0/8 (0.0%)	3/25 (12.0%)	4/30 (13.3%)	3/13 (23.0%)
Colistin	4/118 (3.4%)	0/19 (0.0%)	0/15 (0.0%)	0/11 (0.0%)	0/6 (0.0%)	0/25 (0.0%)	4/29 (13.8%)	0/13 (0.0%)

TMP-SMX: sulfamethoxazole-trimethoprim, TZP: piperacillin-tazobactam, CZA: ceftazidime-avibactam, CZT: ceftolozane-tazobactam, MVB: meropenem-vaborbactam, Q-D: quinupristin-dalfopristin, NT: not tested.

**Table 4:** Lethality rates associated with blood culture positivity for specific pathogens during hospitalization by hospital ward.

Pathogens	All Wards	ICU	Medical Wards	Surgery	NICU
*A. baumannii*	241/396 (60.9%)	183/208 (88.0%)	55/154 (35.7%)	3/34 (8.8%)	0
*S. maltophilia*	23/44 (52.3%)	20/22 (90.9%)	3/16 (18.8%)	0/5 (0.0%)	0/1 (0.0%)
*P. aeruginosa*	108/224 (48.2%)	87/111 (78.4%)	14/81 (17.3%)	7/30 (23.3%)	0/2 (0.0%)
*K. pneumoniae*	282/646 (43.7%)	185/235 (78.7%)	81/311 (26.0%)	10/91 (11.0%)	6/9 (66.7%)
*Serratia* spp.	28/70 (40.0%)	28/38 (73.7%)	0/18 (0.0%)	0/13 (0.0%)	0/1 (0.0%)
*E. faecium*	76/193 (39.4%)	42/50 (84.0%)	32/112 (28.6%)	2/28 (7.1%)	0/3 (0.0%)
*Proteus* spp.	20/57 (35.1%)	11/13 (84.6%)	8/27 (22.9%)	1/9 (11.1%)	0
*E. faecalis*	82/234 (35.0%)	55/69 (79.7%)	22/115 (19.1%)	4/43 (9.3%)	1/7 (14.3%)
*Corynebacterium* spp.	32/100 (32.0%)	15/20 (75.0%)	17/68 (25.0%)	0/11 (0.0%)	0/1 (0.0%)
*S. aureus*	97/398 (24.4%)	48/57 (84.2%)	46/281 (16.4%)	3/55 (5.5%)	0/5 (0.0%)
*Enterobacter* spp.	29/122 (23.8%)	23/30 (76.7%)	4/64 (6.3%)	1/22 (4.5%)	1/6 (16.7%)
*E. coli*	70/380 (18.4%)	38/38 (100%)	29/289 (10.0%)	3/50 (6.0%)	0/3 (0.0%)
*Streptococcus* spp.	13/96 (13.5%)	6/7 (85.7%)	7/73 (9.6%)	0/14 (0.0%)	0/2 (0.0%)

**Table 5:** Multivariable analysis of microorganisms and mortality, adjusted for the results of univariate analysis.

Variables	aOR	CI	*p*
Age	1.030	1.019–1.040	<0.001
Male	0.750	0.602–0.934	0.010
ICU	33.450	24.585–45.512	<0.001
CCI	1.011	0.934–1.094	0.791
Septic shock	5.819	4.203–8.058	0.791
Pneumonia	1.964	1.542–2.502	<0.001
Endocarditis	1.062	0.638–1.769	0.817
CNSI	2.840	1.309–6.162	0.008
COVID-19	1.404	0.799–0.455	0.436
Number of isolates	0.955	0.908–1.006	0.082
*A. baumannii*	1.655	1.143–2.397	0.008
*K. pneumoniae*	1.393	1.032–1.881	0.030
*P. aeruginosa*	1.241	0.760–2.025	0.388
*S. maltophilia*	1.497	0.547–4.092	0.432
Constant	0.017		<0.001

## Data Availability

Data will be made available upon request.

## Supplementary Materials

The following supporting information can be downloaded at: https://www.mdpi.com/article/10.3390/antibiotics14050464/s1, Figure S1. Distribution of the 10 most common isolates per 10,000 patient-days per year. Figure S2. Prevalence of the 10 most common isolates per 10,000 blood cultures. Figure S3. (a) Distribution of microorganisms isolated exclusively from CVCs (n = 638 in 442 patients); (b) frequency of different pathogens in positive CVC blood cultures. Figure S4. Distribution of CVC blood cultures (total), mortality (% Deaths), and CVC-related in-fections/number of positive blood cultures across hospital wards. Figure S5. Survival analysis according to blood isolate microorganism: (a) overall length of stay (LOS), (b) first 14 days.

## Abbreviations

The following abbreviations are used in this manuscript:

BSI	Bloodstream infections
AMR	Antimicrobial resistance
WHO	World Health Organization
ISS	Istituto Superiore di Sanità
MRSA	Methicillin-resistant *S. aureus*
VRE	Vancomycin-resistant *Enterococcus* spp.
IQR	Interquartile range
ICU	Intensive care unit
NICU	Neonatal intensive care unit
UTI	Urinary tract infection
IAI	Intrabdominal infection
CNSI	Central nervous system infection
OI	Osteoarticular infection
SSTI	Skin and soft tissue infection
CCI	Charlson comorbidity index
LOS	Length of stay
CVC	Central venous catheter
TMP-SMX	Trimethoprim-sulfamethoxazole
AMCLI	Associazione microbiologi clinici italiani
EUCAST	European committee on antimicrobial susceptibility testing
CLSI	Clinical and laboratory standard institute
AST	Antimicrobial susceptibility
CI	Confidence interval
cOR	Crude odd ratio
aOR	Adjusted odd ratio
