# Supplementary material for: Outcome of Bloodstream Infections Caused by Antibiotic-Resistant Bacteria: A 7-Year Retrospective Study at the University Hospital of Palermo, Italy
Source: Antibiotics (Basel). 2025 May 1;14(5):464. doi: 10.3390/antibiotics14050464 (PMC12108205; doi:10.3390/antibiotics14050464)
Supplement: Supplementary file 1 [file antibiotics-14-00464-s001.zip › antibiotics-3585275-supplementary.pdf]

antibiotics

MDPI

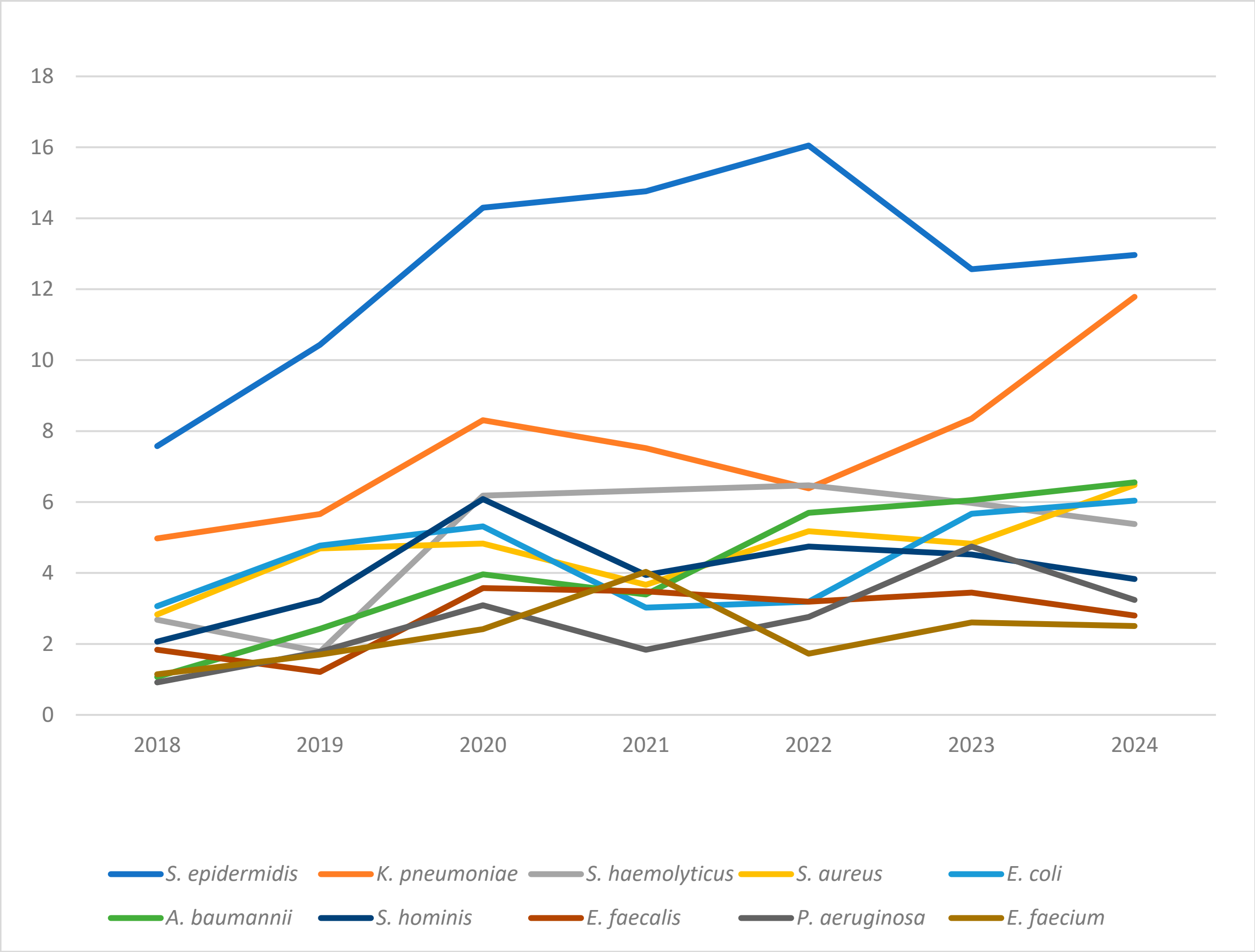

Figure S1. Distribution of the 10 most common isolates per 10,000 patient-days per year.

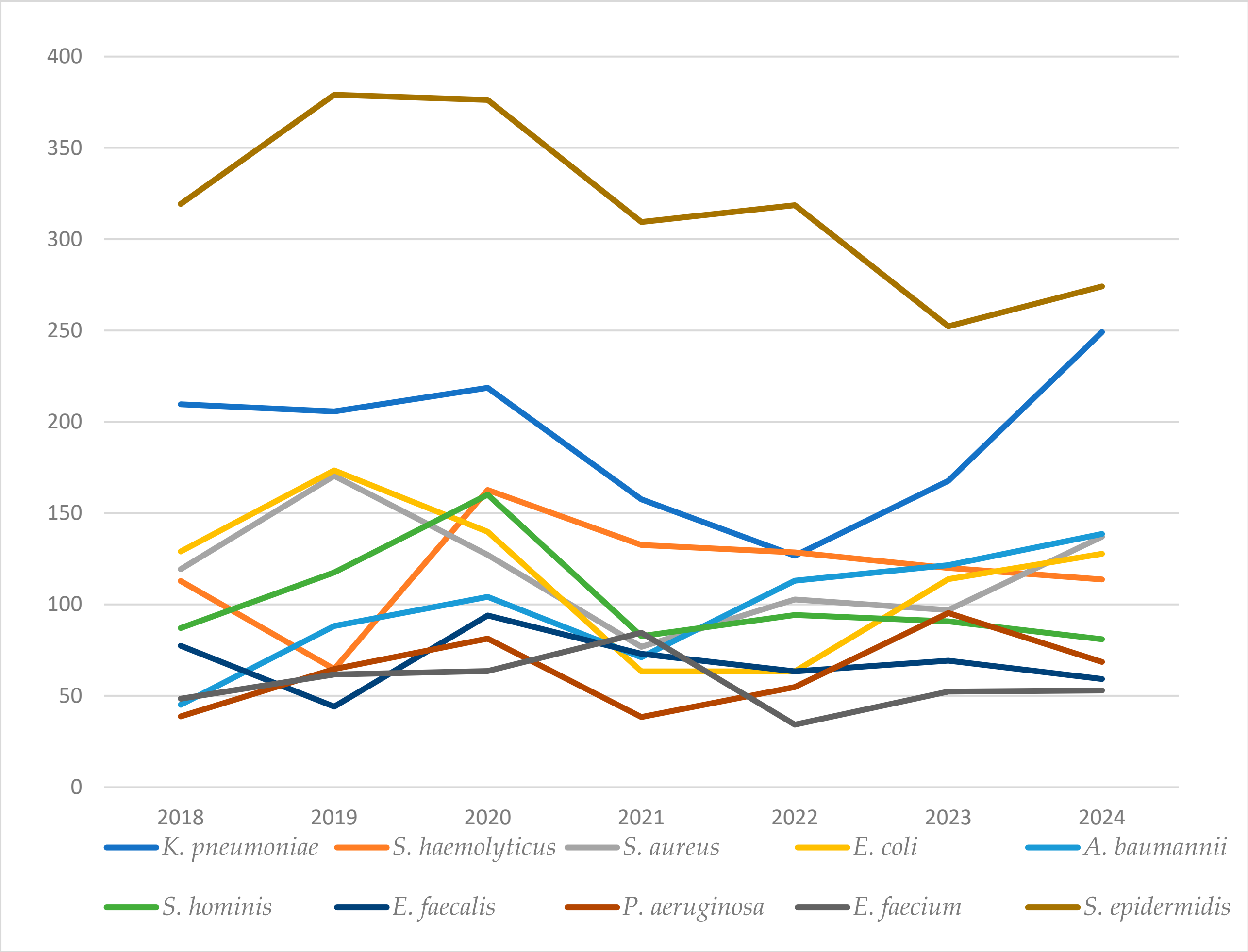

Figure S2. Prevalence of the 10 most common isolates per 10,000 blood cultures.

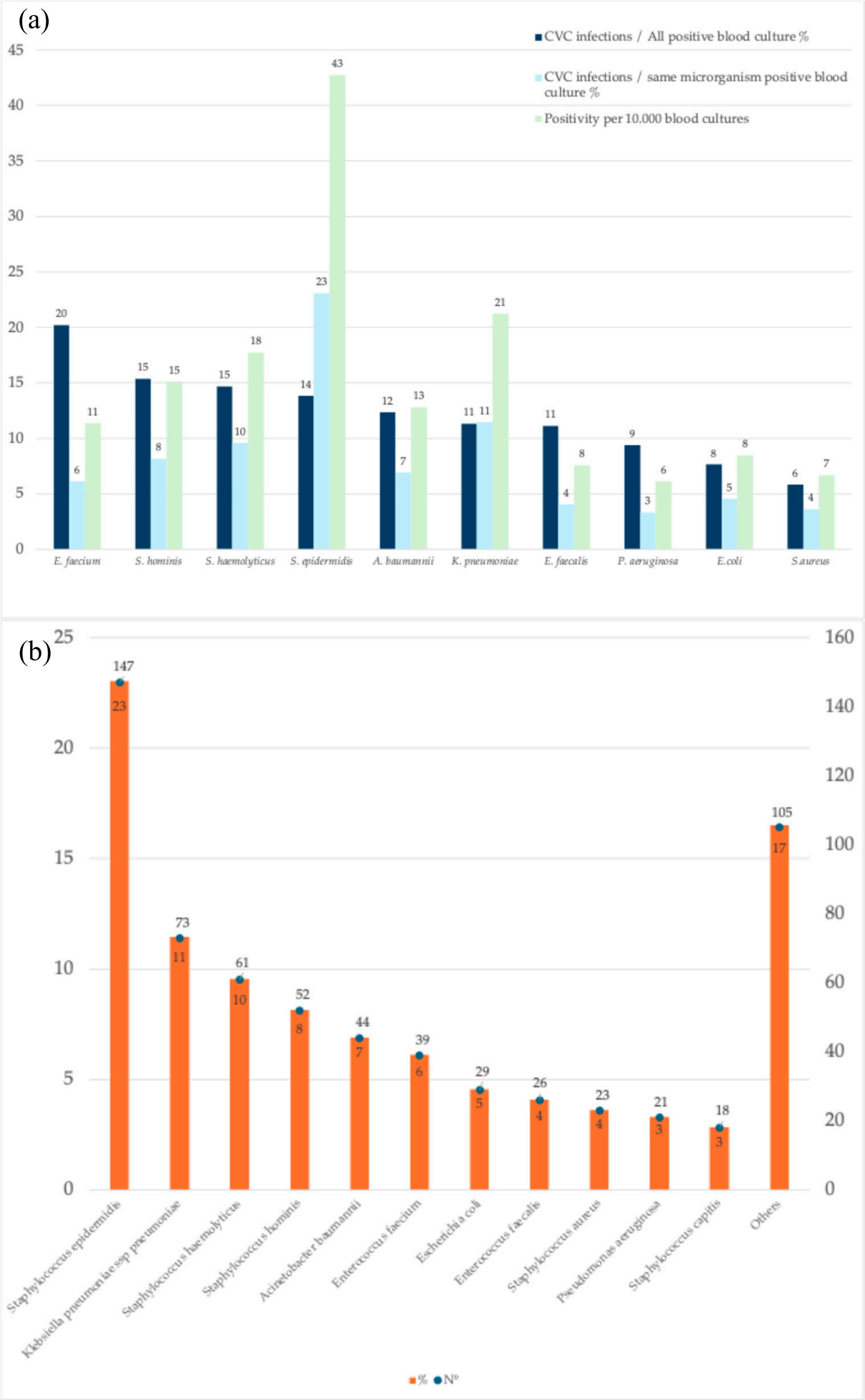

Figure S3. (**a**) Distribution of microorganisms isolated exclusively from CVCs (n =638 in 442 patients); (**b**) frequency of different pathogens in positive CVC blood cultures.

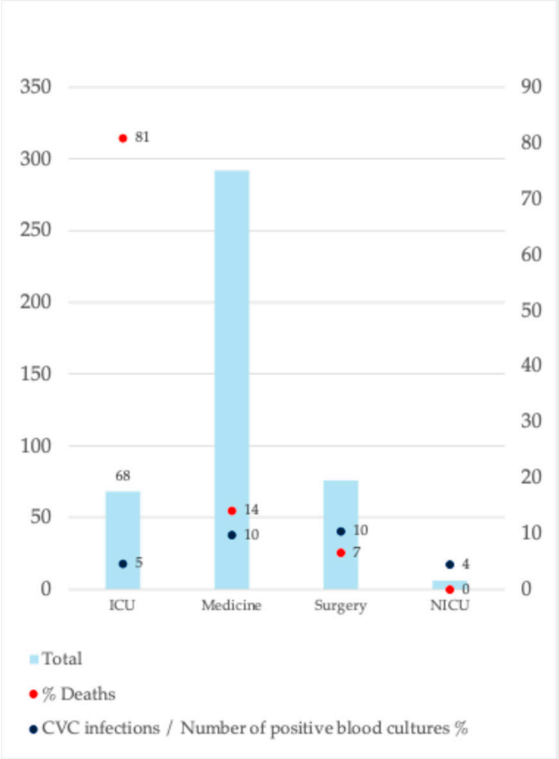

Figure S4. Distribution of CVC blood cultures (total), mortality (% Deaths), and CVC-related infections / number of positive blood cultures across hospital wards.

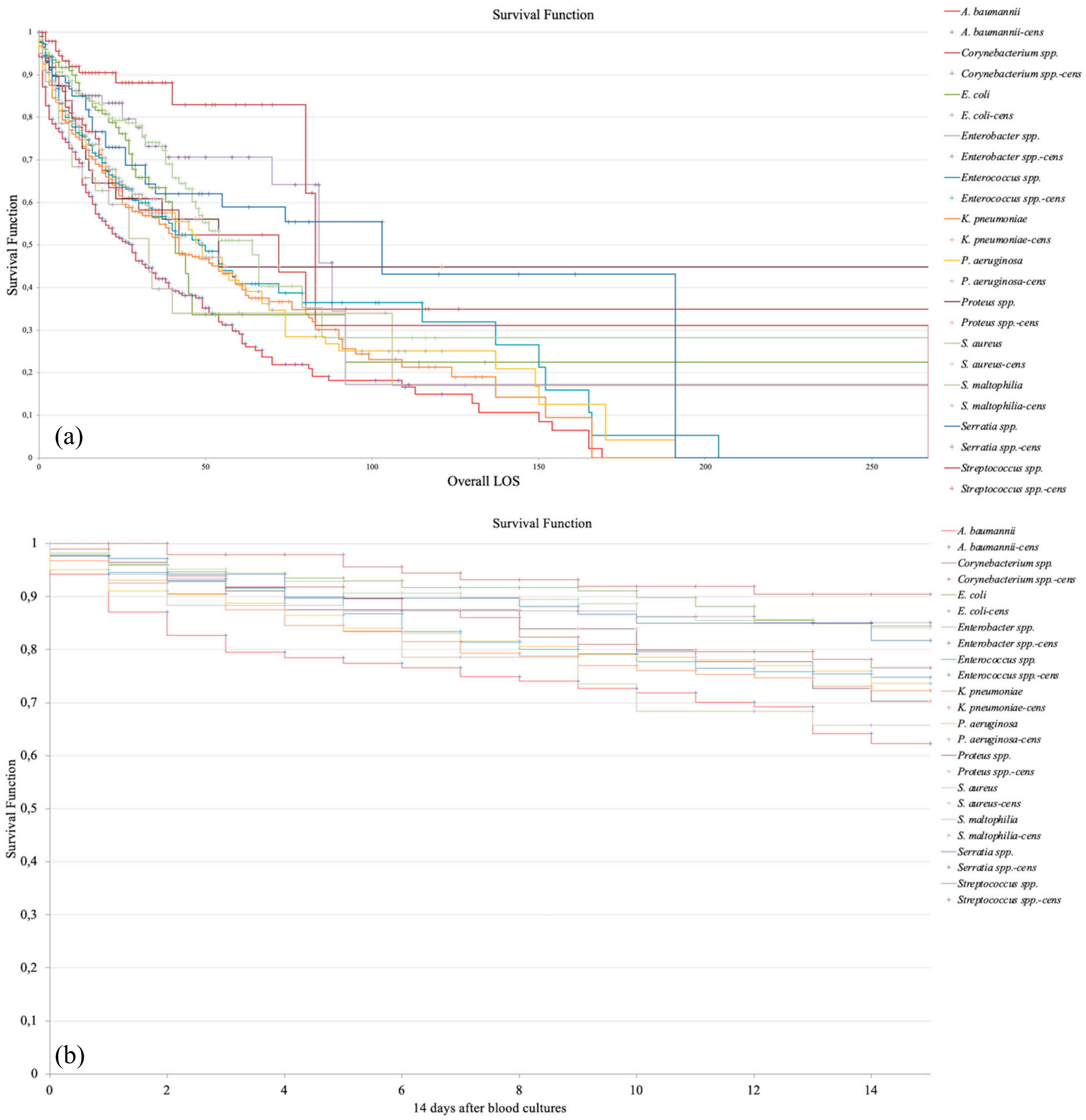

Figure S5. Survival analysis according to blood isolate microorganism: (**a**) overall length of stay (LOS), (**b**) first 14 days.
